# Responses of grapevine cells to physiological doses of ethanol, including induced resistance to heat stress

**DOI:** 10.1111/plb.70064

**Published:** 2025-06-16

**Authors:** A. Diot, G. Madignier, O. Di Valentin, A. Djari, E. Maza, Y. Chen, S. Blanchet, C. Chervin

**Affiliations:** ^1^ Laboratoire de Recherche en Sciences Végétales–Génomique et Biotechnologie des Fruits‐UMR5546, Institut Polytechnique de Toulouse Université de Toulouse, CNRS Toulouse France; ^2^ CNRS, Station d'Ecologie Théorique et Expérimentale (UAR 2029) Moulis France; ^3^ Fondation Jean Poupelain Javrezac France; ^4^ Zhejiang‐Malaysia Joint Research Laboratory for Agricultural Product Processing and Nutrition, College of Food Science and Engineering Ningbo University Ningbo China

**Keywords:** Cell cultures, ethanol, hormesis, RNA‐seq, small heat shock proteins, *Vitis vinifera*

## Abstract

- Grapevine is naturally exposed to stresses like heat, drought, and hypoxia. A recent study found very low oxygen levels inside grape berries, linked to ethanol content. Other studies have established a link between ethanol and tolerance to various stresses: heat, drought and high salinity. The causes of such tolerances are not well understood.
- In our study, 3‐week‐old Gamay calli, *Vitis vinifera*, were characterized for endogenous oxygen and ethanol concentrations. A global transcriptomic study was conducted to explore the response of grapevine cells to ethanol, which, to our knowledge, is the first such analysis in plants. RNA‐seq analysis was performed on cells at 6 and 24 h after treatment with 1 mM ethanol.
- After 6 h, ethanol addition led to 386 differentially expressed genes (DEGs), with notable upregulation of genes related to heat response, especially small Heat Shock Proteins (sHSPs). Further experiments showed that ethanol priming in grape cells or in *Arabidopsis* seedlings reduced pigment and electrolyte leakage under heat stress, respectively.
- This study supports the idea that ethanol priming helps protect plants against heat stress and provides a valuable RNA‐seq dataset for further research into the underlying mechanisms, where sHSPs play a potentially crucial role in this adaptive response.

## Graphical abstract

Ethanol priming enhances heat tolerance in grapevine cells, offering a novel strategy for improving plant resilience to climate stress.

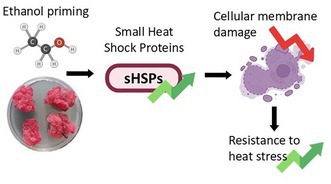

## INTRODUCTION

Hypoxia in plant cells is caused by insufficient oxygen availability (Loreti & Perata 2020). Two types of hypoxia can be distinguished: environmental hypoxia caused by specific environmental cues, and developmental hypoxia in tissues and organs in well‐oxygenated surroundings, under atmosphere oxygen availability (Jethva *et al*. 2022). Environmental hypoxia is periodically encountered by terrestrial plants, often in waterlogged soils (Daniel & Hartman 2024). This can also be induced by rising temperatures, as dissolved oxygen levels decrease at higher temperatures (Chapra *et al*. 2021) and increased microbial activity further depletes oxygen (Alkorta *et al*. 2017). Additionally, hypoxia occurs at high altitudes due to the reduction in oxygen partial pressure (Abbas *et al*. 2022). Developmental hypoxia naturally occurs in bulky or dense tissues like seeds, fruits, teguments and roots, where oxygen diffusion is limited (Brazel & Graciet 2023). It also occurs during rapid cell proliferation when respiration rate is intrinsically high, and oxygen is consumed faster than it can diffuse into plant tissues (Geigenberger 2003). Xiao *et al*. (2018) reported very low levels of oxygen in grape berries during growth and late‐ripening processes. Furthermore, developmental hypoxia has been observed in growing potato tubers (Geigenberger *et al*. 2000), in the embryos of faba bean (*Vicia faba*), in green pea (*Pisum sativum*) (Rolletschek *et al*. 2002) and in seeds during germination (Borisjuk & Rolletschek 2009). Oxygen levels decrease to hypoxic levels during rapid cell proliferation in specific organs, such as the shoot apical meristem and developing monocot anthers in *Zea mays* (Dukowic‐Schulze & van der Linde 2021), and during grapevine bud burst (Meitha *et al*. 2018).

Low oxygen concentrations induce cellular stress related to decreased ATP production, depletion of energy reserves and accumulation of metabolic end‐products, notably ethanol (Jethva *et al*. 2022) through fermentation metabolism (Raymond *et al*. 1995). Ethanol also accumulates during specific plant development stages: in embryos and seedlings, and during fruit development and maturation (Diot *et al*. 2024). Ethanol also accumulates in response to other stresses, including air pollution (sulphur dioxide and ozone exposure), water deficit, and freezing (Kimmerer & Kozlowski 1982).

The impact of ethanol on plant physiology, particularly at low doses, is an emerging area of research as a potential molecular marker of hypoxia (Diot *et al*. 2024). While high concentrations of ethanol are known to be toxic to plants, the few studies that have investigated the effects of low doses of ethanol on plants found beneficial outcomes, such as increased resistance to various stresses. Matsui *et al*. (2022) observed enhanced heat stress resistance in *Arabidopsis thaliana* treated with 60 mM ethanol; Todaka *et al*. (2024) reported heat stress resistance in tomato plants exposed to 20 mM ethanol; Bashir *et al*. (2022) showed that ethanol improved drought resistance across multiple species, including wheat (treated with 50 mM), *Arabidopsis* (5–20 mM ethanol) and rice (100 mM ethanol); and Das *et al*. (2022) found that treatment with 20 mM ethanol conferred resistance to high salinity in faba bean.

Matsui *et al*. (2022) found that exposure to physiological doses of ethanol modulates Heat Shock Protein (HSP) expression in *Arabidopsis* seedlings. HSPs are expressed in response to a wide range of stresses, including thermal, chemical, and oxidative stress (Park & Seo 2015). Among these HSPs, there is a subfamily of small heat shock proteins (sHSPs), which are proposed to act as molecular chaperones to protect other proteins from stress‐induced damage (Waters & Vierling 2020). The relationship between hypoxia and HSPs has already been studied and showed that activation of HSPs is critical to adaptation to hypoxia and enduring the oxidative stress of reoxygenation (Baird *et al*. 2006). Chen *et al*. (2014) further found that HSP70 accumulated more than 10‐fold in the soybean plasma membrane in response to hypoxia. However, none of these studies have established a link between ethanol, hypoxia and HSP. Moreover, most studies on ethanol have investigated response to “hammer” doses of this simple alcohol, i.e. non‐physiological doses.

In the present study, we investigate the impact of ethanol at a physiological dose, 1 mM, on gene transcript expression in grapevine cell cultures, as Xiao *et al*. (2018) found that hypoxic conditions occur during grape berry development. Since the late 1970s, it has been widely accepted that plant cell cultures provide an excellent system for studying cell genetics, physiology, biochemistry and pathology (Smetanska 2008). However, there is a noticeable gap in the literature regarding the characterization of cell cultures grown on solid media. Indeed, most research has focused on plant cell suspension cultures (Hellwig *et al*. 2004; Sato 2013), rather than on plant cell cultures grown on solid agar medium in Petri dishes (i.e. calli).

After characterizing the physiological endogenous concentrations of oxygen and ethanol in these cell cultures, we carried out a whole‐genome transcriptomic study using RNA‐seq. Among the gene families upregulated by a physiological dose of ethanol, we identified genes encoding HSPs. Following this observation, a series of metabolite leakage measurements confirmed the protective role of ethanol priming on grapevine cells exposed to heat shock, as initially described by Matsui *et al*. (2022) in lettuce leaves.

## MATERIAL AND METHODS

### Plant material

Experiments were performed on a grapevine model. Cells from the berry of *Vitis vinifera* L. cv. Gamay were sub‐cultured in 55 × 15 mm Petri dishes using a previously published method (Triantaphylides *et al*. 1993), with modifications concerning the culture medium. The composition of the growth medium used here is provided in Table S1. Parafilm was wrapped around the plates to prevent medium and calli from drying out. The plates were then placed in a climate chamber (CRYO RIVOIRE, Montpellier, France) under constant conditions, with a day/night photoperiod of 16 h/8 h, 25°C/20°C and 150 μmol m^−1^ s^−1^ light. Plates were moved every day within the chamber to ensure homogenous growth conditions.

A complementary experiment was performed on *Arabidopsis thaliana* seedlings var. Col‐0. Seeds were sterilized using 4.8% bleach for 10 min, then sown individually onto 35 × 15 mm Petri dishes containing MS/2 medium with 0, 0.1 or 1 mM ethanol (EtOH), using a sterile toothpick for placement. The Petri dishes were then sealed with micropore surgical tape (3M, 1.25 cm width, Germany) and stratified at 4°C for 24 h. After stratification, the plates were transferred to a growth chamber (CRYO RIVOIRE, France) and cultivated for 11 days under controlled conditions: light/dark, 25°C/20°C, 14 h/10 h and 150 μmol m^−1^ s^−1^. Plates were moved every day within the chamber to ensure homogenous growth conditions.

### Characterization of cell cultures of *V. vinifera* L. cv. Gamay

#### Callus development monitoring

Calli were grown in Petri dishes for 5 weeks and photographed at 0, 7, 14, 21, 28 and 35 days. The selected calli were representative of the observed general development. For each time point, the average callus weight was measured using a microscale. Calli grown for 14 and 35 days were harvested for cross‐sections and photographed using the Axio Zoom.V16 microscope (ZEISS, Jena, Germany).

#### Endogenous ethanol measurements

At different stages, calli were frozen in liquid nitrogen. Each callus was ground to a fine powder and aliquoted at 200 mg sample^−1^. The samples were then thawed in an Eppendorf 5415R centrifuge (Eppendorf, Hamburg, Germany) at 4°C and 13,600×*g* for 10 min. The supernatant was recovered and assayed for ethanol content by measuring NADH absorbance at 340 nm (Ethanol kit; BioSentec, Portet‐sur‐Garonne, France).

#### Endogenous oxygen measurements

Dissolved oxygen in grapevine calli was measured using a Clark O_2_ microelectrode with a 25 μm diameter tip (OX‐25; Unisense A/S, Aarhus, Denmark). The microelectrode was calibrated in 0% oxygen solution (0.1 M NaOH, 0.1 M C_6_H_7_NaO_6_) and in fully oxygenated water (273 μmol L^−1^ at 22°C) as 100% O_2_ solution. An individual callus (equilibrated to room temperature) was placed on a motorized micromanipulator. The microelectrode was placed in the middle of the callus and [O_2_] profiles were taken at depth towards the centre of the callus at 300 μm increments. Oxygen readings were recorded using Unisense Suite software (Unisense A/S). Measurements were performed using 4 calli for each week of development.

### EtOH treatment and sampling for RNA sequencing

**Fig. 1:**
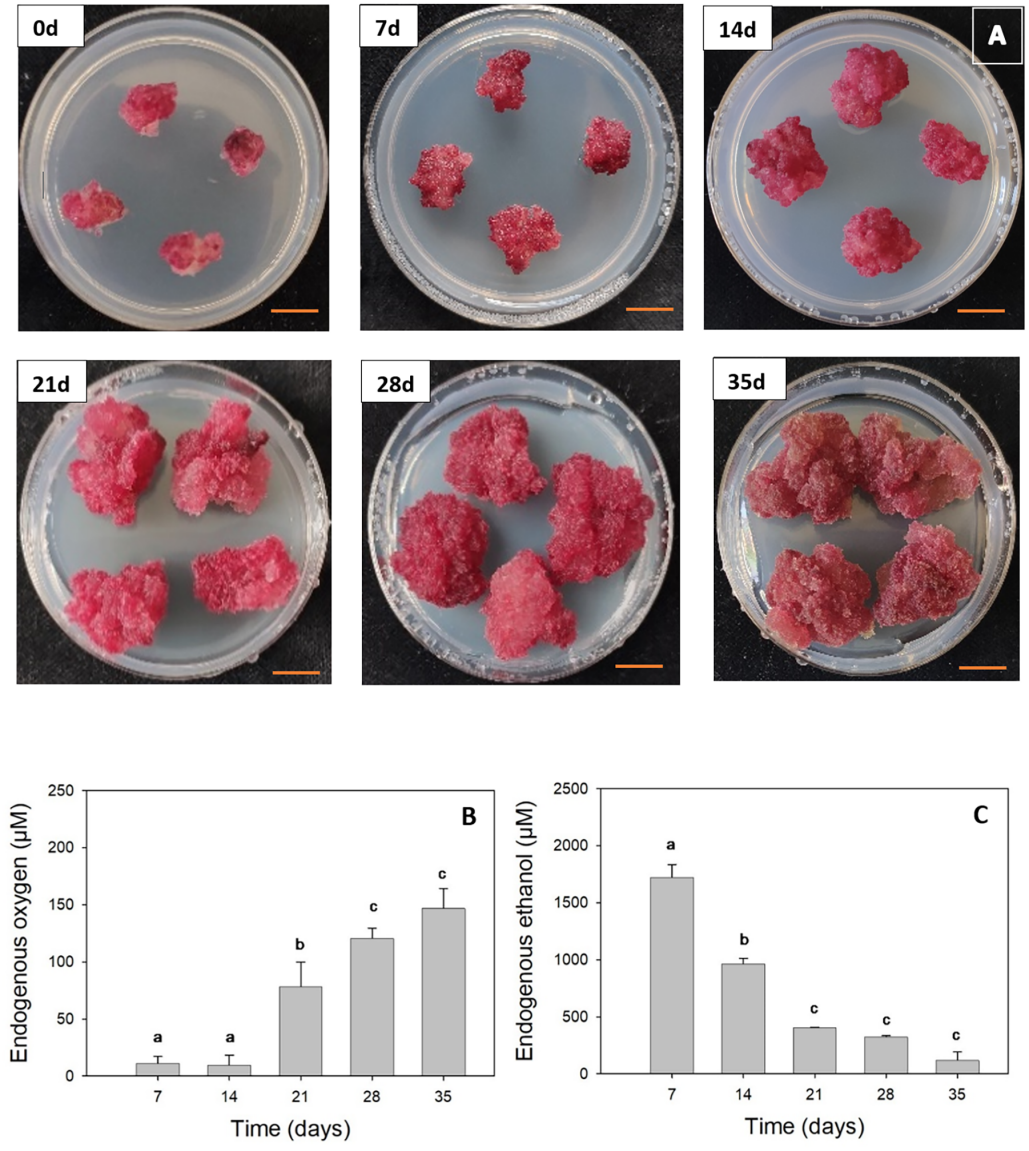
(A) Photographs of Gamay calli at 0 day, 7 days, 14 days, 21 days, 28 days, and 35 days after subculturing onto Petri dishes. The calli selected are representative of the general development observed. Scale: orange bar represents 1 cm. (B) Dissolved O_2_ levels in callus at a distance of 2100 μm towards the callus centre as a function of development time (0, 7, 14, 21, 28 and 35 days), *n* = 4 biological replicates. (C) Ethanol concentration as a function of development time (0, 7, 14, 21, 28 and 35 days), *n* = 3 biological replicates. Error bars represent ± SE and different small letters indicate a significant difference at *P* < 0.05 by multiple comparisons using Fisher's LSD for endogenous oxygen content and Tukey's HSD for endogenous ethanol content.

We selected the callus stage (i.e., 21 days; Fig. 1A) to have sufficient mass of plant material for RNA extractions and because calli were in a healthy growth phase. Considering endogenous ethanol levels in the callus at 21 days (406 ± 2 μM; Fig. 1C), we treated our calli with 1 mM EtOH for the experiment. This treatment represents a 13% increase in ethanol concentration based on average callus weight and the volume of the treatment solution (see Table S2). Due to the limited published research on this subject, two sampling times were chosen: 6 and 24 h post‐treatment, to cover what we called short‐ and long‐term responses. Cell cultures were thus treated with either 100 μL of a control solution (fresh growth medium) or with 100 μL of a fresh growth medium containing 1 mM EtOH, then placed back in the culture chamber for either 6 or 24 h. Growth culture medium for this treatment was prepared without agar. After incubation, each callus was then collected in a 10 mL Falcon™ tube, immediately immersed in liquid nitrogen, then stored at −80°C. In total, the RNA‐seq experiment included 12 samples, consisting of three biological replicates across four conditions: ethanol_0 mM_6 h, ethanol_1 mM_6 h, ethanol_0 mM_24 h, and ethanol_1 mM_24 h.

### RNA extraction and purification

Frozen callus samples were homogenized to powder with a ball mill TissueLyser II (Qiagen, Hilden, Germany) by applying one cycle of 1 min at 250 Hz. Total RNA from individual calli was extracted using 160 mg frozen powder and the Promega ReliaPrep™ RNA Tissue Miniprep System kit (Promega, France; Ref. Z6112) following the manufacturer's protocol, optimized for RNA extractions of grapevine cell cultures (supplementary protocol available on request from the corresponding author). RNAs underwent DNAse treatment with the Invitrogen ezDNase™ Enzyme kit to remove genomic DNA contamination from RNA preparations, following the manufacturer's kit protocol. For each sample, quantity and purity were first assessed by measuring optical density (OD) at 260 nm and 280 nm with a NanoDrop^®^ ND‐1000 UV–Vis spectrophotometer (ThermoFisher, Waltham, MA, USA) and with 1.2% (w/v) gel electrophoresis. Then, for each sample, RNA Integrity Number (RIN) was measured using an Agilent 2100 Bioanalyzer System microfluidic on‐chip capillary electrophoresis device, following the RNA 6000 Nano kit protocol (Agilent Technologies, Waldbronn, Germany).

Samples with a RIN > 8.8 were selected and then aliquoted to obtain 20 μL RNA samples at 50 ng μL^−1^.

### RNA‐seq analyses

Selected RNA samples were sent to Novogene Bioinformatics Technology (Beijing, China) for cDNA library construction and sequencing on the IlluminaNovaSeq 6000 platform (Illumina, San Diego, CA, USA), achieving an average depth of 20 million paired‐end reads per sample. For each of the four conditions, three biological replicates were sent. Raw sequences corresponding to paired‐end reads (2 × 150 bp) were treated as described by Althiab‐Almasaud *et al*. (2021) and Chirinos *et al*. (2023), adapted for the grapevine genome. The raw data discussed here have been deposited in NCBI Gene Expression Omnibus (Edgar *et al*. 2002) and are accessible through GEO Series accession number GSE275842 (https://www.ncbi.nlm.nih.gov/geo/query/acc.cgi?acc=GSE275842). The reads were aligned on the *V. vinifera* L. cv. Chasselas annotated reference genome produced by Djari *et al*. (2024). The quality of these alignments is shown in Table S3A,B.

### Differential expression and downstream analyses

Differential expression (DE) analysis was conducted following the method of Chirinos *et al*. (2023) using R software v. 4.1.1 (https://www.r‐project.org/) and the DESeq2 package v. 1.30.1. More precisely, DE analyses were performed as pairwise comparisons between all pairs of conditions (ex: “etha6 h_1 mM” vs. “etha6 h_0 mM”). The analysis employed the default relative log expression (RLE) normalization method, which accounts for biases caused by library size and the relative size of transcriptomes (Maza *et al*. 2013; Love *et al*. 2014). To control for false positives, the false discovery rate (FDR) was performed using the Benjamini–Hochberg method. Genes were classified as differentially expressed (DEGs) if they had an adjusted *P*‐value (padj) < 0.05 in a multifactorial design analysis. Additional normalization was performed because DESeq2 RLE normalization assumes equal transcript lengths and does not account for transcript length variability. Thus, these normalized data were further adjusted by transcript length to facilitate transcript‐level comparisons in downstream analyses. Such normalized values provide a relative estimation of transcript abundance. The mean expression value of biological replicates was calculated to yield the estimated transcript number for each condition. Principal Components Analysis (PCA) was conducted on raw count data using the plotPCA function in DESeq2, following a variance‐stabilizing transformation to enhance interpretation and reduce noise.

### Identification and annotation of DEGs

The functional annotation of the DEGs was retrieved from the VitExpress transcriptomic platform (Djari *et al*. 2024). When functional annotations were missing, gene ID codes were converted from “Vv” to the taxonomic ID of PNv4 “Vitvi” (Canaguier *et al*. 2017) using the “gene ID converter” tool from GBFwebtools (https://www.grape.resources.gbfwebtools.fr/converter). The “Vitvi” codes were then used to obtain complementary annotations through MapMan Classification (https://mapman.gabipd.org/home) (Schwacke *et al*. 2019). If both annotations were missing, the “Vv” code was used to extract the corresponding gene sequence using “Extract Sequences” (https://www.grape.resources.gbfwebtools.fr/extractSequences) and was subsequently Blasted in NCBI to identify similar sequences and their putative functions (https://blast.ncbi.nlm.nih.gov/Blast.cgi).

### Gene ontology enrichment analysis

Gene ontology (GO) enrichment analysis was performed with R using the enrichGO function from clusterProfiler package v. 4.10.1. Specifically, GO analysis was conducted separately on both the upregulated and downregulated DEG lists obtained from the comparison between EtOH‐treated and control calli. Only DEGs with a |Log2FoldChange| > 2 were selected.

### Ethanol priming and heat‐shock treatments

For *V. vinifera* cv. Gamay cell cultures, 200 μL liquid growth medium with EtOH at different concentrations (0, 0.1, 1, 10, 100 or 1000 mM) was poured onto 3‐week‐old calli under sterile conditions (1 callus/dish). Petri dishes were then sealed with micropore surgical tape (3 M, 1.25 cm width) and returned to the culture chamber for 68 h. After this incubation, optimized by preliminary experiments, heat shock (HS) was applied for 15 min in an incubator in the dark, with a peak temperature of 47°C (recordings of heat stress peak can be found in Fig. S1). The Petri dishes were allowed to cool for 20 min before being returned to the growth chamber. Controls were treated with a control solution (liquid growth medium without EtOH) and were subjected to the heat stress. Double negative controls were treated with a control solution (liquid growth medium without EtOH) and were not subjected to heat stress, but were kept in the dark for the duration of HS.

For *A*. *thaliana* HS treatment, the protocol was adapted from Matsui *et al*. (2022) with the following modifications. *Arabidopsis* seedlings grown for 11 days with 0, 0.1 or 1 mM EtOH were washed with deionized water and placed in test tubes containing 1.8 mL distilled water (15 shoots/tube). The heat stress was applied by placing the tubes in a water bath at 43°C for 30 min then cooling in a water bath at 25°C for 30 min. For each ethanol concentration, controls were performed by maintaining the tubes at 25°C in a water bath for 1 h, without being subjected to heat stress.

### Measurements of cellular leakage after HS treatment

To assess cell damage caused by heat stress in *V. vinifera* and *A. thaliana*, we selected cellular leakage as a key indicator of cell membrane integrity. Cellular leakage is the uncontrolled release of substances, such as ions, metabolites, or macromolecules, from the inside of a cell to its surrounding environment. This phenomenon typically occurs following damage or disruption to the cell membrane (Jiang *et al*. 2015), which normally serves as a selective barrier.

For *Vitis* cell cultures, cellular leakage measurements were assessed by measuring the coloured compounds released into the callus rinsing solution. The protocol was adapted from Ilík *et al*. (2018) with the following modifications: 72 h after HS, calli were harvested, placed in 2 mL Eppendorf tubes, and weighed. To each tube, 1 mL 100 mM mannitol rinsing solution was added to limit osmotic shock. The tubes were then vortexed for 15 s and incubated at room temperature for 20 min on a rotator wheel, followed by centrifugation for 2 min at 16,100×*g*. Absorbance of the supernatant was measured at 420, 520 and 620 nm (Chervin *et al*. 2001; Lambri *et al*. 2015); 100 mM mannitol solution was used as a blank. We report results as relative absorbances, normalized to the HS controls (absorbance values set to 1) to account for variability between batches.

For *Arabidopsis* seedlings, cellular leakage was assessed using the Index of Injury (Id) (Ilík *et al*. 2018), calculated from electrical conductivity measurements. The Id is a quantitative measure used to assess the extent of damage to plant tissues in response to stress, such as temperature extremes. It provides an indication of the degree of cellular or tissue damage, and evaluates the impact of various stressors on plant health. Based on a protocol adapted from Matsui *et al*. (2022), *Arabidopsis* seedlings grown for 11 days were washed with deionized water and placed in test tubes containing 1.8 mL distilled water (15 shoots/tube). Heat stress was applied as described previously. Heat‐stressed and control samples were slowly shaken overnight at 30 rpm, after which electrical conductivity was measured with a conductivity meter (EC‐8801; Prolinx, Dusseldorf, Germany). The samples were then heated for 1 h in a 100°C water bath and shaken at room temperature for several hours, after which the electrical conductivity of the samples was again measured.

### Determination of anthocyanin content

This protocol was adapted from Vitrac *et al*. (2000), which investigated cell suspensions of *V. vinifera* (L.) and, more notably, those of cv. Gamay Teinturier. Some modifications were made: 3‐week‐old calli were treated with 100 μL of either 1 mM EtOH or control solution and incubated for up to 16 days post‐treatment in their growth chamber. Fresh callus tissues were collected, weighed, and placed into 1.5 mL Eppendorf tubes. Each tube received 500 μL solvent mixture composed of methanol and 0.32 M HCl (85:15, v/v). The tissues were ground with a tube pestle for 1 min, followed by incubation for 2 h at 4°C on a rotator wheel. After incubation, the tubes were centrifuged at 16,000×*g* for 2 min. The supernatant was diluted to 1/50th of its original volume with the same solvent.

Absorbance of the anthocyanin extract was measured at 535 nm. Anthocyanin content was calculated using the molar extinction coefficient (log ε = 4.53) reported by Vitrac *et al*. (2000). Methanol and 0.32 M HCl (85:15, v/v) solvent was used as blank.

### Total polyphenol index determination

The Total Polyphenol Index (TPI) is a measure used to quantify the total amount of polyphenolic compounds present in a sample. The TPI method relies on absorption of benzene rings at 280 nm, a characteristic feature of polyphenols. This protocol was adapted from Cetó *et al*. (2012) with the following modification: on the same supernatant extracts as those prepared for anthocyanin determination, absorbance was measured at 280 nm in a quartz cuvette. The TPI for each sample was calculated as the absorbance multiplied by the appropriate dilution factor. Methanol and 0.32 M HCl (85:15, v/v) solvent was used as blank.

### Statistical analyses

All data in Figs. 1 and 4 were analysed with ANOVA and multiple comparison tests, performed with Sigmaplot v. 15 (Inpixon, Palo Alto, CA, USA). The confidence interval was set at 95%, and the *P*‐value to 0.05. Significantly different groups are indicated by small letters (a, b, c) on the graphs. For RNA‐seq data, the statistical analyses were performed using the R software, as specified above.

## RESULTS

### *V. vinifera* L. cv. Gamay cell cultures characterized by hypoxia and physiological ethanol levels in the μM to mM range

Fig. 1A shows grapevine cell cultures as a function of development time over 5 weeks. The increase in callus size throughout development is noticeable, but between days 28 and 35, callus size varied only slightly (for average callus weight over 5‐week development see Fig. S2). Furthermore, the typical colour of our study model, bright pink to light red, is evident in the first 4 weeks of development. A noticeable colour change occurred between days 28 and 35 of development: by day 35, the calli appeared duller compared to those grown for 0, 7, 14, 21 or 28 days.

We then characterized levels of oxygen in grapevine calli during 5 weeks of development on growth medium. Fig. 1B shows dissolved O_2_ levels at 2100 μm towards the callus centre as a function of development time. We found that O_2_ levels increased substantially and gradually during development time, from around 10 μmol L^−1^ in 7‐day‐old calli to 150 μmol L^−1^ in 35‐day‐old calli. Dissolved oxygen levels in calli grown for 7 and 14 days were statistically lower than in 35‐day‐old calli. Complete O_2_ profiles inside calli were measured and sections of 14‐day‐old and 35‐day‐old calli observed, and are provided in Figs. S3 and S4, respectively. There was a strong decrease in ethanol concentration as a function of development time, with an inverse relationship to oxygen levels (Fig. 1C). Ethanol concentration was significantly higher in 7‐day‐old calli than in 35‐day‐old calli, decreasing from 1726 ± 190 μM to 121 ± 99 μM, respectively. Therefore, we can define the range from 100 μM to 1700 μM as “physiological” ethanol concentrations present in cell cultures of *V. vinifera* L. cv. Gamay under normal growth conditions.

### A small exogenous EtOH dose impacts the grapevine transcriptome

**Fig. 2:**
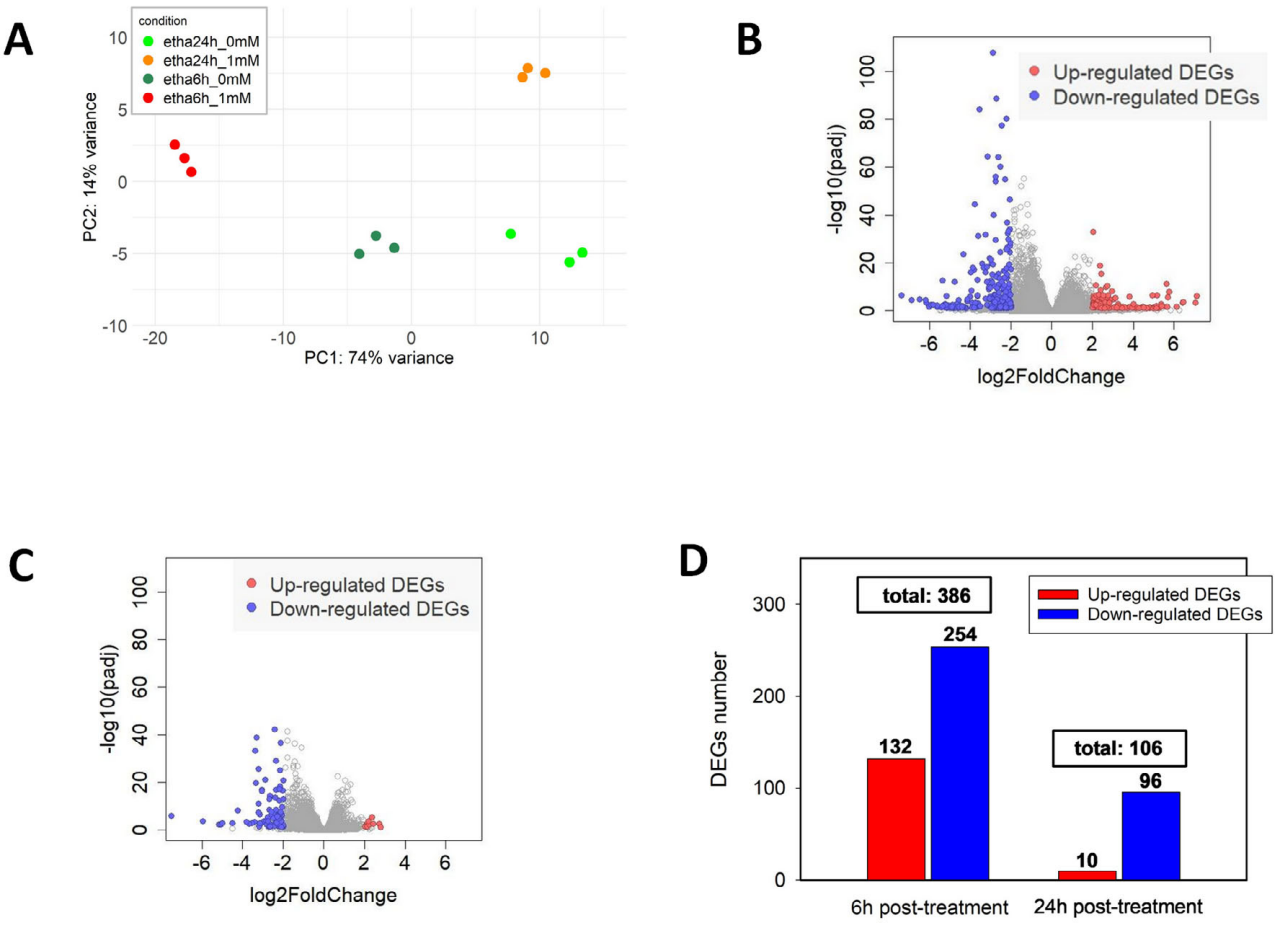
(A) Principal Components Analysis (PCA) of transcriptomic profiles of Gamay grape cell cultures 6 and 24 h after 0 mM control (green and dark green) or 1 mM (orange and red) ethanol treatment, with each symbol representing a replicate. (B) Volcano plot at 6 h post‐treatment. (C) Volcano plot at 24 h post‐treatment. Both plots display differential gene expression for 24,655 genes from cell cultures treated with 1 mM EtOH versus control cultures. Each point represents a gene: the x‐axis shows the log2FoldChange (LFC) comparing EtOH‐treated cells to control cells, while the y‐axis represents the −log10 of adjusted *P*‐values (*P*‐adj), indicating the statistical significance of differential expression. Genes with significant upregulation (LFC ≥ 2 and *P*‐adj ≤ 0.05) are highlighted in red, while significantly downregulated genes (LFC ≤ −2 and *P*‐adj ≤ 0.05) are shown in blue. Genes that did not meet the previous conditions are displayed in grey. (D) Comparative Analysis of DEG number for the comparison between 1 mM EtOH and control, at two time points. The DEGs shown in the figure are any genes with a *P*‐adj ≤ 0.05 and a |LFC| ≥ 2.

To investigate the impact of a physiological dose of EtOH on the *V. vinifera* transcriptome, we employed RNA sequencing (RNA‐seq). In an overview of transcriptional dynamics of the RNA‐seq experiment, the PCA (Fig. 2A) revealed significant differences among the generated transcriptomes, with 74% of the variance associated with PC1 and 14% with PC2. The data consistently demonstrate clustering, wherein transcriptomes align closely according to their respective treatment, forming four distinctive clusters (0 mM_6 h, 1 mM_6 h, 0 mM_24 h, and 1 mM_24 h). This showed that biological variability was smaller than the differences between the four conditions and suggests a discernible impact of a low 1 mM EtOH dose on the transcriptomes. There was more distance between the control and ethanol treatment at 6 h than at 24 h (Fig. 2A). Additionally, it is noteworthy that the 1 mM EtOH treatment at 6 h and at 24 h are furthest apart in the PCA plot, whereas the control solution at 6 and 24 h generated the closest transcriptome groups. There were 5527 DEGs in the 6 h comparison whereas there were 3755 DEGs in the 24 h comparison (Fig. S5).

To further refine our analysis, we applied more stringent criteria to DEGs: we selected genes with a |Log2FoldChange| > 2, in addition to requiring an adjusted *P*‐value < 0.05 (raw results of the Differential Expression Analysis of the comparison 1 vs. 0 mM EtOH at 6 h are available in Table S4). For the comparisons at both 6 and 24 h, the DEG analysis revealed that there was a larger number of downregulated DEGs (Fig. 2C,D). These results show a transcriptional trend towards inhibition, with most genes under‐expressed in ethanol‐treated calli. When visually comparing the number of DEGs between the two post‐treatment durations, the volcano plot for the 6‐h time point shows a larger number of DEGs overall than the plot for the 24‐h comparison (386 vs. 106 genes, respectively; Fig. 2D). These results indicate that even a slight change in ethanol content significantly impacts the grapevine transcriptome.

### Ethanol treatment within the physiological range significantly upregulated small HSP‐related gene expression

**Fig. 3:**
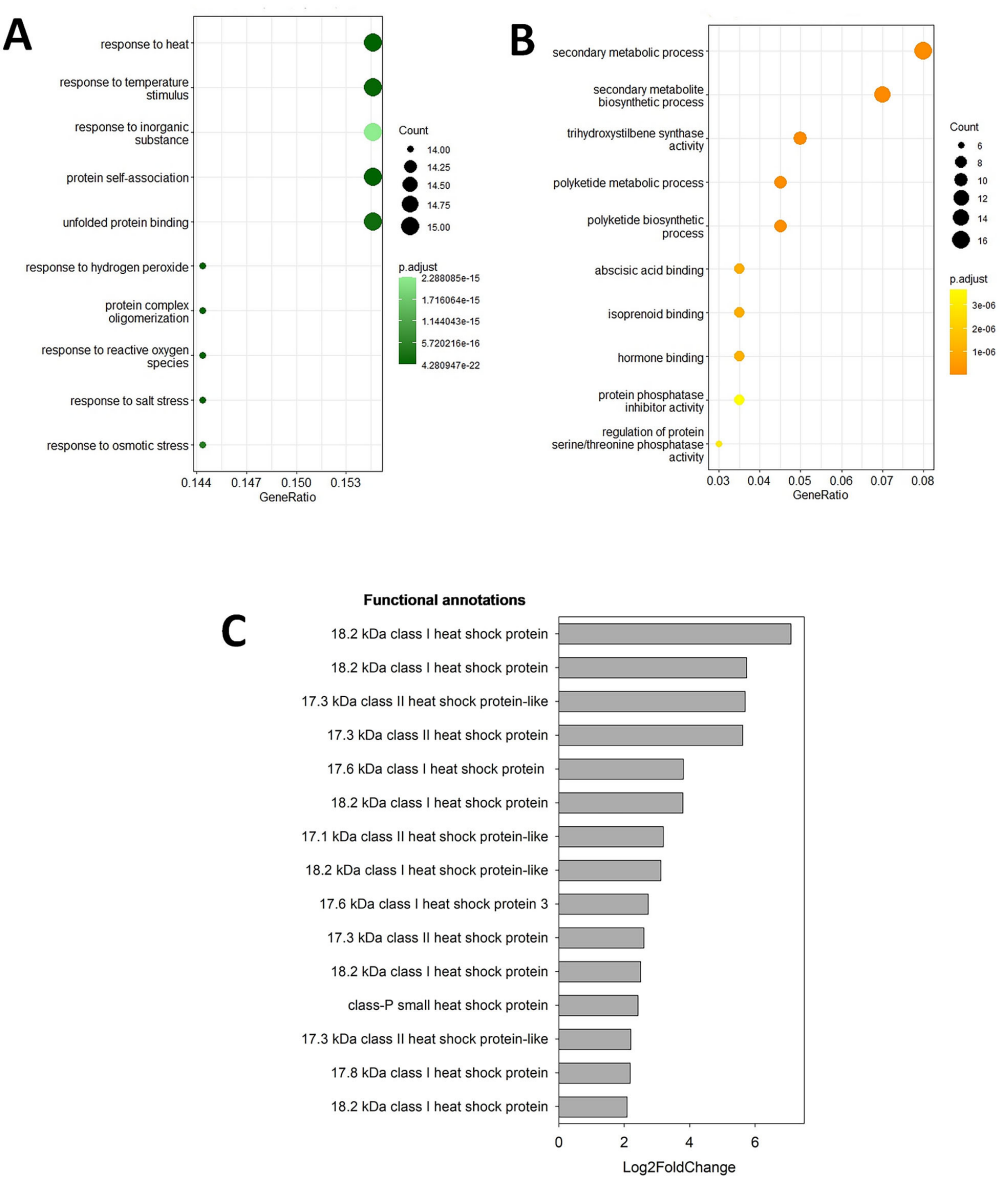
Dot plots of the Gene Ontology (GO) enrichment analysis of (A) upregulated DEGs and (B) downregulated DEGs for the comparison of 1 mM EtOH versus 0 mM at 6 h post‐treatment. DEGs used for the GO enrichment analysis are genes with *P*‐adj < 0.05 and cutoffs as follows: Upregulated DEGs with a Log2FoldChange > 2 and downregulated DEGs with a Log2FoldChange < −2. The size of the dots represents the number of genes in the significant DE gene list associated with the GO term and the colour of the dots represent the enrichment *P*‐adjusted value. (C) Details of the 15 upregulated DEGs within the enriched GO class of “Response to heat” and ordered by their respective Log2 Fold Changes for a 1 mM ethanol treatment compared to control, at 6 h post‐treatment.

Considering the higher number of DEGs at 6 h compared to 24 h post‐treatment, we decided to focus on responses 6 h after treatment. Complete lists of the 386 functionally annotated DEGs are available in Table S5, sorted into two sub‐lists: Table S5 provides downregulated DEGs (254 genes) and Table S5B provides upregulated DEGs (132 genes). Fig. 3A shows results of this GO enrichment for the upregulated DEGs (*P*‐adj < 0.05; Log2FoldChange > 2), while Fig. 3B shows GO enrichment for downregulated DEGs (*P*‐adj < 0.05; Log2FoldChange < −2) (total GO analysis results can be found in Tables S6 and S7).

Genes annotated as “response to heat” (GO:0009408) and “response to temperature stimulus” (GO:0009266), “protein self‐association” (GO:0043621) and “unfolded protein binding” (GO:0051082) were the most represented, with a gene ratio of 0.158, among the 132 upregulated DEGs (Fig. 3A). For downregulated genes, genes annotated as “secondary metabolism process” (GO:0019748) and “secondary metabolite biosynthetic process” (GO:0044550) were most represented among the 254 downregulated DEGs, with gene ratios of 0.08 and 0.07, respectively (Fig. 3B).

We then examined the specific genes comprising the enriched families in both DEGs lists. First, we examined specific genes in the upregulated enriched family associated with the GO term “Response to heat”. This list included 15 DEGs (Fig. 3C; respective gene IDs, functional annotations, Log2FoldChange values, and adjusted *P*‐values in Table S8). For functional annotations in Fig. 3C, all upregulated DEGs were small Heat Shock Proteins (sHSP) and fell into two major groups: 9 cytosolic class I sHSP and 5 cytosolic class II sHSP. The last gene is a class‐P sHSP. The Log2FoldChanges of these DEGs ranged from 2.09 to 7.09 with very low associated *P*‐adj values (0.039 to 5.19^−12^), providing a high level of confidence that ethanol treatment is responsible for this differential expression.

We then examined the specific genes comprising the downregulated enriched family associated with the GO term “Secondary metabolism process”. This list included 16 DEGs (Fig. S6) with the respective gene IDs, functional annotations, Log2FoldChange values, and adjusted *P*‐values; Table S9. Functional annotations (Fig. S6) included genes involved in the general phenylpropanoid pathway, e.g. phenylalanine ammonia‐lyase (PAL), and genes involved in phenylpropanoid downstream pathways. Indeed, genes involved in pathways for stilbenoid and flavonoid biosynthesis, such as stilbene synthase (STS) and chalcone synthase (CHS), were downregulated. The Log2FoldChanges associated with these DEGs ranged from −2.02 to 4.90, being less extreme than those associated with “Response to heat” genes.

### Ethanol‐priming reduced heat‐induced cell leakage

**Fig. 4:**
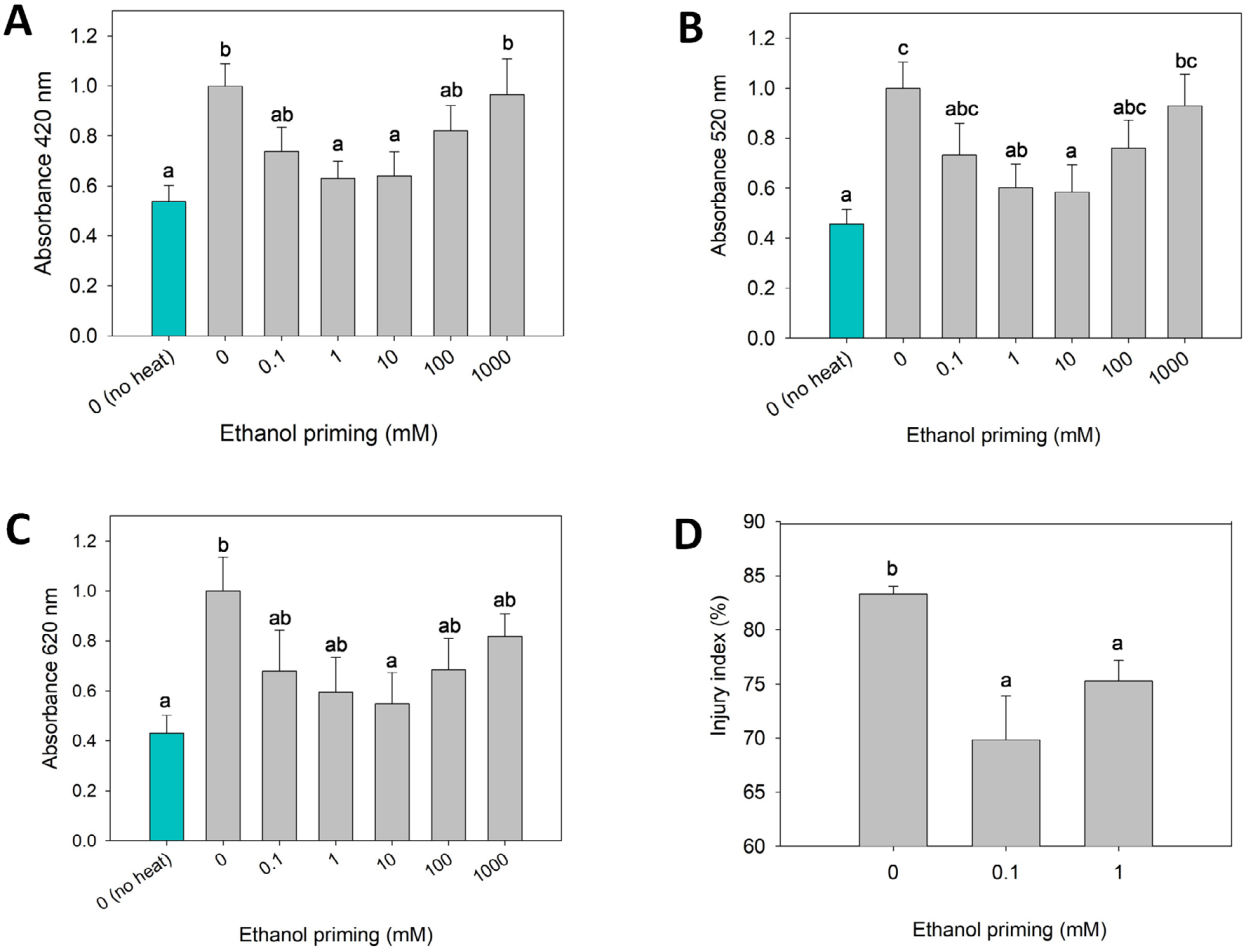
Damage induced by heat stress in *Vitis vinifera* Gamay cell cultures and *Arabidopsis thaliana* Col‐0 seedlings, as a function of pretreatments with various ethanol concentrations. Pigments released from Gamay cell cultures, absorbing at (A) 420 nm, yellow range, (B) 520 nm, red range, and (C) 620 nm, blue range, following a 47°C heat‐stress (see Material and Methods for details). Results are mean ± SE, *n* = 8 biological replicates, small letters indicate a significant difference (*P* < 0.05) between groups, multiple comparisons by Fisher's LSD. (D) Injury index of *Arabidopsis* seedlings based on ion leakage measurements following a 43°C heat‐stress (see Material and Methods for details). Results mean ± SE, *n* = 7 biological replicates, small letters indicate a significant difference (*P* < 0.05) between groups, multiple comparisons by Tukey's HSD.

Following the observation that 1 mM EtOH significantly upregulated sHSPs in the GO analysis, we looked for thermotolerance phenotypes in ethanol‐primed calli after heat stress. We chose a 10‐fold increment for ethanol concentration to analyse the dose–response effect. This allowed us to extend the concentration range around 1 mM, thus accommodating potential variations in the phenotype (Fig. 4). The double negative controls, without ethanol priming or exposure to heat stress, had the lowest absorbances at 420, 520 and 620 nm (cyan bars, Fig. 4A–C), showing basal cell leakage. In contrast, cell cultures exposed to both the control pre‐treatment (0 mM EtOH) and HS had the highest absorbance at all three wavelengths. Calli primed with EtOH from 0.1 to 10 mM, displayed a continuous decreasing trend in absorbance at 420, 520, and 620 nm, compared to the controls. Absorbance increased again in calli primed with 100 and 1000 mM EtOH. These inverted bell‐shaped curves were observed across all three monitored absorbances.

Overall, calli primed with 1 and 10 mM EtOH showed strong reductions in absorbance of 35%–45% compared with the HS control. This shows that priming grapevine cell cultures with low physiological doses of ethanol (i.e., 0.1 to 10 mM) reduce cell leakage after exposure to a mild heat shock. Moreover, a complementary experiment on *A. thaliana* seedlings to confirm that low doses of EtOH limit cellular leakage (Fig. 4D) found that, in control seedlings, the injury index (83%) was significantly higher than in seedlings treated with 0.1 nM (69%) and 1 mM (75%) EtOH. This corresponds to a significant reduction in the injury index of 14% for the 0.1 mM ethanol treatment and 8% for the 1 mM ethanol treatment compared to the control. This shows that priming *Arabidopsis* seedlings with low physiological doses of ethanol significantly reduced ion released after exposure to a mild heat shock, and further reinforces the results described for the grapevine cell cultures.

### Ethanol‐priming does not impact polyphenol content in grapevine calli

As shown in Fig. 3B, the GO terms ‘secondary metabolic process’ and ‘secondary metabolite biosynthetic process’ were significantly enriched among the 254 downregulated DEGs, many of which are linked to polyphenol synthesis (see Fig. S6). Given this significant downregulation in secondary metabolism, and particularly downregulated PAL and CHS transcripts, we investigated polyphenol content in ethanol‐primed calli. We monitored anthocyanin and total polyphenol content as a function of time in grapevine cell cultures, following an initial 1 mM EtOH treatment, and found changes in anthocyanin concentration and total polyphenol index in cell extracts (Fig. S7). Both graphs show an increasing trend as a function of time, however, there was no significant difference in anthocyanin concentrations (Fig. S7A) or in total polyphenol index (Fig. S7B) between ethanol‐primed and control treatments.

## DISCUSSION

### Grapevine cell cultures, a model experiencing hypoxia and ethanol accumulation

Xiao *et al. (* 2018) found low levels of oxygen in grape berries and even anoxia in the centre of the berries, similar to our current findings, with developmental hypoxic to anoxic oxygen levels inversely correlated with ethanol concentrations in the calli, especially during rapid growth in the first 2 weeks after subculturing (Fig. 1B,C). Rapid growth is associated with a high rate of cell division and high cellular oxygen consumption in most plant tissues (Raymond *et al*. 1995). The increase in oxygen levels in calli, as they age, is probably linked to a decrease in their growth and metabolic rates. This slower growth allows oxygen to diffuse faster than it is consumed by the cells. Additionally, after 5 weeks of growth, cross‐sections (Fig. S4) revealed heterogeneous structure with invaginations and gaseous interstices within the callus, further facilitating air and oxygen diffusion. There is a close relationship between oxygen and ethanol levels in plants, in which decreased oxygen results in increased ethanol concentration, due to a shift from aerobic to anaerobic respiration (Bui *et al*. 2019). Therefore, we conclude that our cell culture model naturally experiences developmental hypoxia, leading to ethanol accumulation. This validates the cell culture model for studying the impact of small changes in ethanol concentrations. These could be related to climate change, as rising temperatures will reduce dissolved oxygen in water and in organisms, thus increasing respiration rates. These changes lead to hypoxic metabolism in certain organisms and tissues, and to ethanol production. The question arises whether ethanol serves as a signal prompting organisms to adapt, or if it is simply an end‐product of hypoxic metabolism (Diot *et al*. 2024).

### Low exogenous applications of EtOH trigger a rapid response in the grapevine transcriptome and cells perceive it as stress

We found small changes in ethanol concentration generate rapid responses at the transcriptome level in Gamay cell cultures. In the PCA plot (Fig. 2A), the closely grouped transcriptomes based on their respective treatments and the high explained variance demonstrate the significant impact of ethanol on gene transcripts. The sum of the variances (PC1 + PC2), which accounted for 88% of the total variance, indicates that the two‐dimensional plot effectively represents the impact of the ethanol treatment and the two sampling time points (6 and 24 h). It is possible that the highly controlled *in vitro* culture environment and the use of identical cloned cells limited biological variability in the experiments. Gamay cell cultures sensed very small changes in ethanol concentration (1 mM), as evidenced by the 386 DEGs identified at 6 h (Fig. 2D), suggesting that a small amount of ethanol is rapidly detected by the cells and prompts a strong and immediate transcriptional response. The reduction in number of DEGs to 106 at 24 h post‐treatment (Fig. 2D) shows that the transcriptional response diminished over time. This is further reinforced by the decreased number of DEGs with a high Log2FoldChange after 24 h compared to 6 h (Fig. 2C vs. Fig. 2B). This trend suggests that the transcriptional response to low doses of ethanol is not only strong and immediate but also relatively transient. The general trend of downregulation of the transcriptomic dynamics when comparing 1 mM EtOH to control treatments (Fig. 2D), at 6 and 24 h post‐treatment, is often observed in plant responses to stress. Indeed, Ul *et al*. (2019) demonstrated that a common plant strategy to cope with stressful situations is to reduce synthesis of normal proteins, and enhance production of stress‐related proteins, such as HSPs. This trade‐off is clearly illustrated at the transcriptomic level in our study: Gamay calli downregulate genes involved in secondary metabolism (Fig. 3B) while simultaneously overexpressing genes related to stress proteins following addition of exogenous ethanol (Fig. 3A). GO revealed that cell sHSP genes were upregulated in calli treated with 1 mM EtOH compared to the control at 6 h (Fig. 3A). sHSPs function as early responders to assist cells in managing proteins destabilized by stress (Janowska *et al*. 2019), and their induction represents a rapid and intense emergency response (Parsell & Lindquist 1993). This aligns with our RNA‐seq data where, just 6 h after treatment with 1 mM EtOH, expression of some sHSP genes increased more than 64‐fold compared with the control (Fig. 3C). Our study suggests that a minor change in ethanol is sensed by cells as a stress, prompting them to adapt. However, we did not measure changes in ethanol concentration in the calli between the treatment and RNA extraction time points. Such changes could result from callus metabolism (e.g. oxidation, carbon assimilation) or from evaporation, with each factor potentially influencing DEGs. These aspects should be explored in future studies. However, it is important to note that the DEGs found in our study can be primarily attributed to the ethanol treatment.

### Response of grapevine cells to physiological ethanol levels: Upregulation of small heat shock protein transcripts

The genes associated with the GO terms “Response to heat” and “Response to temperature stimulus” were the most statistically overrepresented 6 h after a 1 mM EtOH treatment, notably sHSPs (Fig. 3C). First, the ability of ethanol at physiological doses to stimulate sHSP synthesis has been observed in the animal kingdom, with studies finding that ethanol at 50 mM induced sHSP genes in mouse embryonic neural stem cells (Choi *et al*. 2011) and EtOH at 60 mM increased levels of HSP27 in mouse cortical neurons (Pignataro *et al*. 2007).

#### The sHSPs

HSPs are classified into five subfamilies based on molecular weight: Hsp100, Hsp90, Hsp70, Hsp60, and sHSPs (Sun & MacRae 2005). The sHSPs are differentiated by their low molecular weight, typically plant sHSPs are 15 to 30 kDa. The proteins of this subfamily are most abundant in plants (Vierling 1991), where they are expressed during normal development (Lindquist & Craig 1988), and known to respond to environmental stresses, including heat, cold, drought, salinity and oxidative stress. The high diversification of plant sHSPs reflects molecular adaptation to stress. sHSPs have evolved to be sensitive to small changes in pH or metal ions near physiological concentrations (Janowska *et al*. 2019), hence it is likely that they are also sensitive to other small changes near‐physiological state, in our case, small changes in ethanol concentration. sHSPs are particularly known for their role in maintaining proteostasis in response to cellular stress (Haslbeck & Vierling 2015), specializing in preventing protein aggregation by binding to partially unfolded proteins under stress (Lee *et al*. 1995, 1997; McLoughlin *et al*. 2016; Janowska *et al*. 2019), which differentiates them from larger HSPs, as sHSPs typically do not refold proteins. Besides this, sHSPs can function as membrane stabilizers and ROS scavengers, or act synergistically with antioxidant systems. Overall, they play a key role in maintaining membrane fluidity and permeability under some stresses (Aghdam *et al*. 2013). However, ethanol modifies the fluidity of phospholipid bilayers (Patra *et al*. 2006), possibly creating a link between ethanol, membrane organization, and induction of sHSPs.

#### Class CI and CII sHSPs

In Fig. 3C the key genes were sHSP genes encoding class CI and CII subfamilies. These are the two most abundant classes of plant cytosolic‐localized sHSPs. CI sHSPs comprise the largest family of sHSP genes in higher plants (Waters 2013), while the CII gene family is smaller. Basha *et al*. (2010) examined structural and functional differences between CI and CII subfamilies and found that both the CI and CII subfamilies have chaperone activity and effectively prevent irreversible aggregation, illustrating their role in prevention of future cell damage. Therefore, it is not surprising that 6 h after the EtOH treatment, there was a significant upregulation of these protein transcripts. We hypothesize that the plant uses very small changes in ethanol to prevent further stress damage. Indeed, ethanol induced sHSP in soybean (Kuo *et al*. 2000) and rice (Guan *et al*. 2004), even at doses of 5% (v/v), corresponding to nearly 1 M, which can be considered a “hammer” dose.

### Small changes in ethanol concentration confer thermotolerance to grapevine calli by reducing cell leakage, as confirmed using *Arabidopsis* seedlings

Our model is a cell culture rich in anthocyanins (Guan *et al*. 2016; Kong *et al*. 2021), which led us to examine release of pigments following heat stress (Lambri *et al*. 2015). Zhang *et al*. (2005) showed that heat treatment (38°C for 10 h) caused ultrastructural injury to *Vitis* mesophyll cells, which was linked to increases in conductivity associated with membrane leakage, and to an increase in malondialdehyde. In oenology, postharvest heat treatment of grape berries increases berry skin cell damage and facilitates release of anthocyanins (Lambri *et al*. 2015). Lambri *et al*. (2015) showed that heating grape berries at temperatures from 60 to 80°C for 3 to 60 min increased absorbances at 420, 520 and 620 nm, which define red wine colour. After preliminary experiments, we chose a rather mild heat treatment (Fig. S1) to illustrate differences induced by ethanol priming. The results showed (Fig. 4A–C) significant differences in pigment leakage and that ethanol priming protected grapevine calli from heat stress damage. This protective effect has also been found in other plant species, as here in *Arabidopsis* seedlings, where electrical conductivity indicated that ethanol priming protected the seedlings from heat stress damage (Fig. 4D). This suggests that the ethanol‐induced‐sHSPs are related to acquisition of thermotolerance. Several authors have found a strong correlation between sHSP accumulation and plant tolerance to stress (Wang *et al*. 2004; Liu *et al*. 2012), suggesting that the protective effect involves membrane stabilization. According to Ilík *et al*. (2018), heat acclimation is connected to an increase in degree of saturation of fatty acids in membrane lipids, leading to increased rigidity of cell membranes and increased thermostability; although there was no evidence of desaturase‐related‐genes in our RNA‐seq data. However, Tsvetkova *et al*. (2002) suggested that the association between sHSPs and membranes may constitute a general mechanism that preserves membrane integrity during thermal fluctuations.

Plants have evolved many mechanisms for adaptation to dynamic and adverse environmental conditions. They have also acquired a form of ‘stress memory’, retaining the response to an initial stress that can prime responses to a second stress, allowing a rapid and strong response (Savvides *et al*. 2016), as well as ability produce compounds that induce expression of stress‐related genes, whose products protect cellular components from abiotic stress (Sako *et al*. 2020). This is called chemical priming, and is evident in our results, as primed cell cultures gave better protection against mild heat stress. Todaka *et al*. (2024) treated tomato plants with 20 mM ethanol and found enhanced heat stress tolerance, highlighting the potential of ethanol‐based priming as a technology to mitigate heat stress damage in crops. Crosstalk between heat and ethanol stresses, and heat and ethanol tolerance, has been found in several species and seems to be a conserved trait across kingdoms (Matsui *et al*. 2022). Similar results are found in other kingdoms: HSP induction in mammals (Chinese hamster; Li 1983), and in the microorganism, *Saccharomyces cerevisiae* (Plesset *et al*. 1982).

*Timeframe of responses*: sHSP expression is often part of an immediate stress response (Hu *et al*. 2022) but their synthesis is not maintained through time (Altschuler & Mascarenhas 1982). Our results reflect this, as no sHSP genes were upregulated 24 h post‐ethanol treatment. However, 68 h after ethanol priming, thermotolerance phenotypes were observed (Fig. 4A–C). Vierling (1991) reported that pea plants exposed to heat stress accumulated chloroplastic sHSPs and class CI HSPs, even after the stress had ended. The half‐life of these proteins was around 52 h for chloroplastic sHSPs and 37 h for cytoplasmic HSPs. This shows that although expression of sHSPs is transient, the sHSPs themselves are quite stable. Their persistence suggests that their participation in recovery could be as important as their possible role during the stress period itself (Vierling 1991). However, in the present study the priming effect in mitigating heat stress only when the stress was mild. Indeed, when the stress exceeds a certain threshold (higher than sublethal heat shock), the priming effect diminishes, and both treated cells and *Arabidopsis* seedlings exhibit increased leakage compared to controls (data not shown).

### Response of grapevine to physiological doses of ethanol: Downregulation of key polyphenol‐related transcripts, without effecting anthocyanin and polyphenol content

The downregulation of secondary metabolism is a classic plant response to stress (Bilgin *et al*. 2010; Liu *et al*. 2012; Ul *et al*. 2019). However, there was no significant difference between EtOH treated and untreated calli in anthocyanin or total polyphenol content (Fig. S7). We suggest two main hypotheses to explain the difference at transcriptomic and physiological levels. First, because anthocyanins and polyphenols are very important in Gamay calli, transient downregulation of enzymes involved in biosynthesis of polyphenols is very small. Indeed, the Gamay aux is a teinturier grape with rare and unique red‐fleshed berries and red skin, which is uncommon in grape cultivars which generally have a red skin but white flesh (Guan *et al*. 2016). Second, the polyphenol pathway is complex with numerous proteins involved. The fact that the expression of some of these protein transcripts is inhibited does not ensure that polyphenolic compounds accumulate at different levels.

### Plant response to ethanol displays hormesis, and ethanol might be a messenger of hypoxia or other stresses

Because the sHSP subfamily was extremely overexpressed in the RNA‐seq experiment, and to examine physiological response of cell cultures to low doses of ethanol, we assessed cell damage from a heat shock in calli primed with different ethanol concentrations. The results (Fig. 4A–C) clearly show a biphasic dose–response curve for the absorbances tested, which is typical of an hormesis effect. Hormesis is where exposure to low doses of a stressor or toxin result in a beneficial effect on the organism, contrary to expected harmful effects at higher doses (Calabrese & Baldwin 2002, 2003). This concept challenges the traditional linear dose–response model by proposing U‐shaped or inverse U‐shaped response curves (Erofeeva 2022). According to Calabrese & Baldwin (2002, 2003), hormesis is an adaptive response of organisms to stimuli (environment, chemicals, …). At low doses, the stressor triggers adaptive responses within cells and organisms, such as increased antioxidant production (Wang *et al*. 2023) and repair mechanisms (Scott 2008), which enhance resilience and improve overall health. These authors suggest that this could be the result of compensatory biological processes, following an initial disruption in homeostasis, i.e. in our study, the slight change in ethanol concentration. Although there is no single biological mechanism that can explain hormesis across plants, Agathokleous *et al*. (2020) suggested certain general principles of mechanisms of hormesis across different species and stress‐inducing agents: low doses of stress induce a mild increase in production of reactive chemical species, hormones, and enzyme and non‐enzyme antioxidants, as well as upregulation of proteins (e.g., heat shock proteins), which lead to reduced toxicity if plants are subsequently exposed to severe stress within a preconditioning framework. Our study found both an increase in HSP transcripts (Fig. 3B) and reduced HS toxicity (Fig. 4) after ethanol treatment. This hormesis effect of ethanol was described by Middleton *et al*. (1978) on the architecture of pea roots, where mean root length was increased by low ethanol (0.002 mL.L^−1^, equivalent to 0.0002% or 0.034 mM). More recently Bhattacharya *et al*. (1985) showed that ethanol increased root formation in mung bean, with a maximum at 0.2% (equivalent to 34.25 mM), and Wu *et al*. (2019) reported that ethanol improved both biomass (at 0.0125–0.05 mL.L^−1^, equivalent to 0.21–0.86 mM) and micronutrient accumulation in oilseed rape. The curve shapes of cellular leakage observed in our study (Fig. 4), on two different plant species, add to these previous results. Our results suggest that ethanol at physiological concentrations interacts with grapevine and *Arabidopsis* biological systems, where small amounts activate adaptive and beneficial responses, while larger amounts overwhelm these systems, leading to toxicity. Given its potential role in activating adaptive responses, we hypothesize that ethanol may serve as a signalling molecule in plants and could be a potential messenger for stress.

Further research is needed to explore ethanol‐induced hormesis in plants, specifically to elucidate how stimulation and inhibition vary with ethanol concentration. While the inhibition observed at high concentrations is expected, as it aligns with the onset of toxicity, the stimulation effect at low concentrations warrants more detailed investigation. Considering ethanol as a small molecule with significant diffusive capacity, notably through membranes, and considering its hydrophobic nature, the manner by which cells perceive small variations in ethanol concentration remains a matter of research and debate (Diot *et al*. 2024).

## CONCLUSIONS

We demonstrated that grapevine cells can sense ethanol at a physiological concentration, inducing a strong and rapid transcriptomic response, typical for stressors. This transcriptomic response has physiological repercussions, as evidenced by thermotolerance biphasic dose–response curves, suggesting that ethanol could act as a molecular messenger in stressful situations in plants, rather than just being an end‐product metabolite of hypoxic metabolism. Harnessing this response could be promising in preparing plants for more challenging stress. Indeed, plants biosynthesize phytohormones and other metabolites to adapt to adverse environments. Recent studies have demonstrated that exogenous treatment with chemical compounds enhance abiotic stress tolerance by inducing molecular and physiological defence mechanisms, a process known as chemical priming (Sako *et al*. 2020). The development of effective strategies that mitigate plant abiotic stress is essential for sustainable agriculture and food security, especially with continuing global population growth. Chemical priming represents a promising strategy for mitigating abiotic stress in crop plants. Given its low cost and environmental non‐persistence, we propose that ethanol could be a good candidate for plant chemical priming and a valuable tool in agriculture, especially for mitigating environmental stress impacts on crops.

## AUTHOR CONTRIBUTIONS

CC, YC and AD developed the project concept and research design. AD performed the experiments. GM and AD performed the RNA‐seq data cleaning, reads alignment and quality assessment of reads. AD, EM and GM performed analyses in RODV, GM and AD made the R graphs. AD and CC wrote the original draft. All co‐authors contributed to editing the manuscript. AD, SB and CC were involved in funding acquisition, CC and SB were involved in AD PhD supervision.

## FUNDING INFORMATION

This study was supported by the École Universitaire de Recherche TULIP‐GS (ANR‐18‐EURE‐0019), which provided half of a PhD grant to A. Diot and a full PhD grant to Olivia D.V. All authors are also grateful to the Occitanie Region for funding the other half of the PhD grant to A. Diot. The research was also partly funded by the VitiFunGen project supported by the Fondation Jean Poupelain (Cognac, France), the Labex TULIP (ANR‐10‐LABX‐41) and by OxyFruit ANR (ANR‐23‐CE20‐0001). Additionally, we thank the Université Paul Sabatier, CNRS, and Toulouse INP for their contributions to our research.

## CONFLICT OF INTEREST STATEMENT

The authors declare they have no conflicts of interest.

## Supporting information


**Fig. S1.** Heat stress monitoring in Gamay grape cell cultures.
**Fig. S2.** Average weight of a callus of *Vitis vinifera* cv. Gamay as a function of development time after sub‐culturing.
**Fig. S3.** Dissolved O_2_ profiles of *Vitis vinifera* L. cv. Gamay cell cultures as a function of development time.
**Fig. S4.** Photographs of calli from above and photographs of calli cross‐sections.
**Fig. S5.** Transcriptional dynamics, showing number of DEGs upregulated and downregulated, 6 and 24 h after a 1 mM ethanol treatment, compared to controls.
**Fig. S6.** Details of the 16 DEGs within the enriched GO term “Secondary metabolism process”.
**Fig. S7.** (A and B) Monitoring anthocyanin concentration and total polyphenol index of Gamay cell cultures after an initial 1 mM ethanol treatment or control treatment.
**Table S1.** Composition of the Gamay cell culture growth medium.
**Table S2.** The ‘concentration‐effect’ of ethanol treatment in 3‐week‐old calli, at the time of treatment with exogenous ethanol.
**Table S3.** (A and B) Quality of alignment and Assignment rate to features (RNA‐seq data).

**Table S4.** Results of Differential Expression Analysis comparing 0 versus 1 mM EtOH at 6 h.

**Table S5.** Lists of 386 functionally annotated DEGs for the 6 h comparison, 1 mM versus 0 mM: 254 downregulated DEGs (A) and 132 upregulated DEGs (B).

**Table S6.** Overall GO enrichment analysis results for the upregulated DEG list (132 genes).

**Table S7.** Overall GO enrichment analysis results for the downregulated DEG list (254 genes).

**Table S8.** Details of upregulated DEGs within the enriched GO term “Response to heat”, resulting from the GO enrichment analysis.

**Table S9.** Details of downregulated DEGs within the enriched GO term “Secondary metabolism process”, resulting from the GO enrichment analysis.

## Data Availability

The raw data underlying this article have been deposited in NCBI's Gene Expression Omnibus (Edgar *et al*. 2002) and are accessible through GEO Series accession number GSE275842 (https://www.ncbi.nlm.nih.gov/geo/query/acc.cgi?acc=GSE275842).

## References

[plb70064-bib-0001] Abbas M. , Sharma G. , Dambire C. , Marquez J. , Alonso‐Blanco C. , Proaño K. , Holdsworth M.J. (2022) An oxygen‐sensing mechanism for angiosperm adaptation to altitude. Nature, 606, 565–569. 10.1038/s41586-022-04740-y 35650430 PMC9200633

[plb70064-bib-0002] Agathokleous E. , Kitao M. , Calabrese E.J. (2020) Hormesis: highly generalizable and beyond laboratory. Trends in Plant Science, 25, 1076–1086. 10.1016/j.tplants.2020.05.006 32546350

[plb70064-bib-0003] Aghdam M.S. , Sevillano L. , Flores F.B. , Bodbodak S. (2013) Heat shock proteins as biochemical markers for postharvest chilling stress in fruits and vegetables. Scientia Horticulturae, 160, 54–64. 10.1016/j.scienta.2013.05.020

[plb70064-bib-0004] Alkorta I. , Epelde L. , Garbisu C. (2017) Environmental parameters altered by climate change affect the activity of soil microorganisms involved in bioremediation. FEMS Microbiology Letters, 364, fnx200. 10.1093/femsle/fnx200 28961781

[plb70064-bib-0005] Althiab‐Almasaud R. , Chen Y. , Maza E. , Djari A. , Frasse P. , Mollet J.C. , Mazars C. , Jamet E. , Chervin C. (2021) Ethylene signaling modulates tomato pollen tube growth through modifications of cell wall remodeling and calcium gradient. The Plant Journal, 107, 893–908. 10.1111/tpj.15353 34036648

[plb70064-bib-0006] Altschuler M. , Mascarenhas J.P. (1982) Heat shock proteins and effects of heat shock in plants. Plant Molecular Biology, 1, 103–115. 10.1007/BF00024974 24317892

[plb70064-bib-0007] Baird N.A. , Turnbull D.W. , Johnson E.A. (2006) Induction of the heat shock pathway during hypoxia requires regulation of heat shock factor by hypoxia‐inducible factor‐1. The Journal of Biological Chemistry, 281, 38675–38681. 10.1074/jbc.M608013200 17040902

[plb70064-bib-0008] Basha E. , Jones C. , Wysocki V. , Vierling E. (2010) Mechanistic differences between two conserved classes of small heat shock proteins found in the plant cytosol. Journal of Biological Chemistry, 285, 11489–11497. 10.1074/jbc.M109.074088 20145254 PMC2857027

[plb70064-bib-0009] Bashir K. , Todaka D. , Rasheed S. , Matsui A. , Ahmad Z. , Sako K. , Utsumi Y. , Vu A.T. , Tanaka M. , Takahashi S. , Ishida J. (2022) Ethanol‐mediated novel survival strategy against drought stress in plants. Plant and Cell Physiology, 63, 1181–1192. 10.1093/pcp/pcac114 36003026 PMC9474946

[plb70064-bib-0010] Bhattacharya S. , Bhattacharya N.C. , Bhatnagar V.B. (1985) Effect of ethanol, methanol and acetone on rooting etiolated cuttings of Vigna radiata in presence of sucrose and auxin. Annals of Botany, 55, 143–145. 10.1093/oxfordjournals.aob.a086885

[plb70064-bib-0011] Bilgin D.D. , Zavala J.A. , Zhu J.I.N. , Clough S.J. , Ort D.R. , DeLucia E.H. (2010) Biotic stress globally downregulates photosynthesis genes. Plant, Cell & Environment, 33, 1597–1613. 10.1111/j.1365-3040.2010.02167.x 20444224

[plb70064-bib-0012] Borisjuk L. , Rolletschek H. (2009) The oxygen status of the developing seed. New Phytologist, 182, 17–30. 10.1111/j.1469-8137.2008.02752.x 19207684

[plb70064-bib-0013] Brazel A.J. , Graciet E. (2023) Complexity of abiotic stress stimuli: mimicking hypoxic conditions experimentally on the basis of naturally occurring environments, Plant abiotic stress signaling. Springer, New York, USA, pp 23–48. 10.1007/978-1-0716-3044-0_2 36944871

[plb70064-bib-0014] Bui L.T. , Novi G. , Lombardi L. , Iannuzzi C. , Rossi J. , Santaniello A. , Mensuali A. , Corbineau F. , Giuntoli B. , Perata P. , Zaffagnini M. (2019) Conservation of ethanol fermentation and its regulation in land plants. Journal of Experimental Botany, 70, 1815–1827. 10.1093/jxb/erz052 30861072 PMC6436157

[plb70064-bib-0015] Calabrese E.J. , Baldwin L.A. (2002) Defining hormesis. Human and Experimental Toxicology, 21, 91–97. 10.1191/0960327102ht217oa 12102503

[plb70064-bib-0016] Calabrese E.J. , Baldwin L.A. (2003) Ethanol and hormesis. Critical Reviews in Toxicology, 33, 407–424. 10.1080/713611043 12809430

[plb70064-bib-0017] Canaguier A. , Grimplet J. , Di Gaspero G. , Scalabrin S. , Duchêne E. , Choisne N. , Mohellibi N. , Guichard C. , Rombauts S. , Le Clainche I. , Berard A. (2017) A new version of the grapevine reference genome assembly (12X.v2) and of its annotation (VCost.v3). Genomics Data, 14, 56. 10.1016/j.gdata.2017.09.002 28971018 PMC5612791

[plb70064-bib-0018] Cetó X. , Gutiérrez J.M. , Gutiérrez M. , Céspedes F. , Capdevila J. , Mínguez S. , Jiménez‐Jorquera C. , Del Valle M. (2012) Determination of total polyphenol index in wines employing a voltammetric electronic tongue. Analytica Chimica Acta, 732, 172–179. 10.1016/j.aca.2012.02.026 22688049

[plb70064-bib-0019] Chapra S.C. , Camacho L.A. , McBride G.B. (2021) Impact of global warming on dissolved oxygen and BOD assimilative capacity of the world's rivers: modeling analysis. Watermark, 13, 2408. 10.3390/w13172408

[plb70064-bib-0020] Chen Y. , Chen X. , Wang H. , Bao Y. , Zhang W. (2014) Examination of the leaf proteome during flooding stress and the induction of programmed cell death in maize. Proteome Science, 12, 33. 10.1186/1477-5956-12-33 25028572 PMC4099015

[plb70064-bib-0021] Chervin C. , Elkereamy A. , Roustan J.P. , Faragher J.D. , Latché A. , Pech J.C. , Bouzayen M. (2001) An ethanol spray at veraison enhances colour in red wines. Australian Journal of Grape and Wine Research, 7, 144–145. 10.1111/j.1755-0238.2001.tb00202.x

[plb70064-bib-0022] Chirinos X. , Ying S. , Rodrigues M.A. , Maza E. , Djari A. , Hu G. , Liu M. , Purgatto E. , Fournier S. , Regad F. , Bouzayen M. (2023) Transition to ripening in tomato requires hormone‐controlled genetic reprogramming initiated in gel tissue. Plant Physiology, 191, 610–625. 10.1093/plphys/kiac464 36200876 PMC9806557

[plb70064-bib-0023] Choi M.R. , Jung K.H. , Park J.H. , Das N.D. , Chung M.K. , Choi I.G. , Lee B.C. , Park K.S. , Chai Y.G. (2011) Ethanol‐induced small heat shock protein genes in the differentiation of mouse embryonic neural stem cells. Archives of Toxicology, 85, 293–304. 10.1007/s00204-010-0591-z 20871982

[plb70064-bib-0024] Daniel K. , Hartman S. (2024) How plant roots respond to waterlogging. Journal of Experimental Botany, 75, 511–525. 10.1093/jxb/erad332 37610936

[plb70064-bib-0025] Das A.K. , Anik T.R. , Rahman M.M. , Keya S.S. , Islam M.R. , Rahman M.A. , Sultana S. , Ghosh P.K. , Khan S. , Ahamed T. , Ghosh T.K. (2022) Ethanol treatment enhances physiological and biochemical responses to mitigate saline toxicity in soybean. Plants, 11, 272. 10.3390/plants11030272 35161252 PMC8838166

[plb70064-bib-0026] Diot A. , Groth G. , Blanchet S. , Chervin C. (2024) Responses of animals and plants to physiological doses of ethanol: a molecular messenger of hypoxia? FEBS Journal, 291, 1102–1110. 10.1111/febs.17056 38232057

[plb70064-bib-0027] Djari A. , Madignier G. , Di Valentin O. , Gillet T. , Frasse P. , Djouhri A. , Hu G. , Julliard S. , Liu M. , Zhang Y. , Regad F. (2024) Haplotype‐resolved genome assembly and implementation of VitExpress, an open interactive transcriptomic platform for grapevine. Proceedings of the National Academy of Sciences of the United States of America, 121, e2403750121. 10.1073/pnas.2403750121 38805269 PMC11161759

[plb70064-bib-0028] Dukowic‐Schulze S. , van der Linde K. (2021) Oxygen, secreted proteins and small RNAs: mobile elements that govern anther development. Plant Reproduction, 34, 1–19. 10.1007/s00497-020-00401-0 33492519 PMC7902584

[plb70064-bib-0029] Edgar R. , Domrachev M. , Lash A.E. (2002) Gene expression omnibus: NCBI gene expression and hybridization array data repository. Nucleic Acids Research, 30, 207–210. 10.1093/nar/30.1.207 11752295 PMC99122

[plb70064-bib-0030] Erofeeva E.A. (2022) Hormesis in plants: its common occurrence across stresses. Current Opinion in Toxicology, 30, 100333. 10.1016/j.cotox.2022.02.006

[plb70064-bib-0031] Geigenberger P. (2003) Response of plant metabolism to too little oxygen. Current Opinion in Plant Biology, 6, 247–256. 10.1016/s1369-5266(03)00038-4 12753974

[plb70064-bib-0032] Geigenberger P. , Fernie A.R. , Gibon Y. , Christ M. , Stitt M. (2000) Metabolic activity decreases as an adaptive response to low internal oxygen in growing potato tubers. Biological Chemistry, 381, 723–740. 10.1515/BC.2000.093 11030430

[plb70064-bib-0033] Guan J.C. , Jinn T.L. , Yeh C.H. , Feng S.P. , Chen Y.M. , Lin C.Y. (2004) Characterization of the genomic structures and selective expression profiles of nine class I small heat shock protein genes clustered on two chromosomes in rice (*Oryza sativa* L.). Plant Molecular Biology, 56, 795–809. 10.1007/s11103-004-5182-z 15803416

[plb70064-bib-0034] Guan L. , Dai Z. , Wu B.H. , Wu J. , Merlin I. , Hilbert G. , Renaud C. , Gomès E. , Edwards E. , Li S.H. , Delrot S. (2016) Anthocyanin biosynthesis is differentially regulated by light in the skin and flesh of white‐fleshed and teinturier grape berries. Planta, 243, 23–41. 10.1007/s00425-015-2391-4 26335854

[plb70064-bib-0035] Haslbeck M. , Vierling E. (2015) A first line of stress defense: small heat shock proteins and their function in protein homeostasis. Journal of Molecular Biology, 427, 1537–1548. 10.1016/j.jmb.2015.02.002 25681016 PMC4360138

[plb70064-bib-0036] Hellwig S. , Drossard J. , Twyman R.M. , Fischer R. (2004) Plant cell cultures for the production of recombinant proteins. Nature Biotechnology, 22, 1415–1422. 10.1038/nbt1027 15529167

[plb70064-bib-0037] Hu C. , Yang J. , Qi Z. , Wu H. , Wang B. , Zou F. , Mei H. , Liu J. , Wang W. , Liu Q. (2022) Heat shock proteins: biological functions, pathological roles, and therapeutic opportunities. MedComm, 3, e161. 10.1002/mco2.161 35928554 PMC9345296

[plb70064-bib-0038] Ilík P. , Špundová M. , Šicner M. , Melkovičová H. , Kučerová Z. , Krchňák P. , Fürst T. , Večeřová K. , Panzarová K. , Benediktyová Z. , Trtílek M. (2018) Estimating heat tolerance of plants by ion leakage: a new method based on gradual heating. New Phytologist, 218, 1278–1287. 10.1111/nph.15097 29573424

[plb70064-bib-0039] Janowska M.K. , Baughman H.E. , Woods C.N. , Klevit R.E. (2019) Mechanisms of small heat shock proteins. Cold Spring Harbor Perspectives in Biology, 11, a034025. 10.1101/cshperspect.a034025 30833458 PMC6771367

[plb70064-bib-0040] Jethva J. , Schmidt R.R. , Sauter M. , Selinski J. (2022) Try or die: dynamics of plant respiration and how to survive low oxygen conditions. Plants, 11, 205. 10.3390/plants11020205 35050092 PMC8780655

[plb70064-bib-0041] Jiang X. , Feng K. , Yang X. (2015) In vitro antifungal activity and mechanism of action of tea polyphenols and tea saponin against Rhizopus stolonifer. Journal of Molecular Microbiology and Biotechnology, 25, 269–276. 10.1159/000430866 26138353

[plb70064-bib-0042] Kimmerer T.W. , Kozlowski T.T. (1982) Ethylene, ethane, acetaldehyde, and ethanol production by plants under stress. Plant Physiology, 69, 840–847. 10.1104/pp.84.4.1204 16662306 PMC426315

[plb70064-bib-0043] Kong J. , Wu J. , Guan L. , Hilbert G. , Delrot S. , Fan P. , Liang Z. , Wu B. , Matus J.T. , Gomès E. , Dai Z. (2021) Metabolite analysis reveals distinct spatio‐temporal accumulation of anthocyanins in two teinturier variants of cv. ‘Gamay’ grapevines (*Vitis vinifera* L.). Planta, 253, 1–18. 10.1007/s00425-021-03613-4 33788027

[plb70064-bib-0044] Kuo H.F. , Tsai Y.F. , Young L.S. , Lin C.Y. (2000) Ethanol treatment triggers a heat shock‐like response but no thermotolerance in soybean (Glycine max cv. Kaohsiung No. 8) seedlings. Plant, Cell & Environment, 23, 1099–1108. 10.1046/j.1365-3040.2000.00621.x

[plb70064-bib-0045] Lambri M. , Torchio F. , Colangelo D. , Segade S.R. , Giacosa S. , De Faveri D.M. , Gerbi V. , Rolle L. (2015) Influence of different berry thermal treatment conditions, grape anthocyanin profile, and skin hardness on the extraction of anthocyanin compounds in the colored grape juice production. Food Research International, 77, 584–590. 10.1016/j.foodres.2015.08.027

[plb70064-bib-0046] Lee G.J. , Pokala N. , Vierling E. (1995) Structure and in vitro molecular chaperone activity of cytosolic small heat shock proteins from pea. Journal of Biological Chemistry, 270, 10432–10438. 10.1074/jbc.270.18.10432 7737977

[plb70064-bib-0047] Lee G.J. , Roseman A.M. , Saibil H.R. , Vierling E. (1997) A small heat shock protein stably binds heat‐denatured model substrates and can maintain a substrate in a folding‐competent state. EMBO Journal, 16, 659–671. 10.1093/emboj/16.3.659 9034347 PMC1169668

[plb70064-bib-0048] Li G.C. (1983) Induction of thermotolerance and enhanced heat shock protein synthesis in Chinese hamster fibroblasts by sodium arsenite and by ethanol. Journal of Cellular Physiology, 115, 116–122. 10.1002/jcp.1041150203 6841458

[plb70064-bib-0049] Lindquist S. , Craig E.A. (1988) The heat‐shock proteins. Annual Review of Genetics, 22, 631–677. 10.1146/annurev.ge.22.120188.003215 2853609

[plb70064-bib-0050] Liu G.T. , Wang J.F. , Cramer G. , Dai Z.W. , Duan W. , Xu H.G. , Wu B.H. , Fan P.G. , Wang L.J. , Li S.H. (2012) Transcriptomic analysis of grape (*Vitis vinifera* L.) leaves during and after recovery from heat stress. BMC Plant Biology, 12, 1–10. 10.1186/1471-2229-12-174 23016701 PMC3497578

[plb70064-bib-0051] Loreti E. , Perata P. (2020) The many facets of hypoxia in plants. Plants, 9, 745. 10.3390/plants9060745 32545707 PMC7356549

[plb70064-bib-0052] Love M.I. , Huber W. , Anders S. (2014) Moderated estimation of fold change and dispersion for RNA‐seq data with DESeq2. Genome Biology, 15, 1–21. 10.1186/s13059-014-0550-8 PMC4302049 25516281

[plb70064-bib-0053] Matsui A. , Todaka D. , Tanaka M. , Mizunashi K. , Takahashi S. , Sunaoshi Y. , Tsuboi Y. , Ishida J. , Bashir K. , Kikuchi J. , Kusano M. (2022) Ethanol induces heat tolerance in plants by stimulating unfolded protein response. Plant Molecular Biology, 110, 131–145. 10.1007/s11103-022-01291-8 35729482

[plb70064-bib-0054] Maza E. , Frasse P. , Senin P. , Bouzayen M. , Zouine M. (2013) Comparison of normalization methods for differential gene expression analysis in RNA‐seq experiments: a matter of relative size of studied transcriptomes. Communicative & Integrative Biology, 6, e25849. 10.4161/cib.25849 26442135 PMC3918003

[plb70064-bib-0055] McLoughlin F. , Basha E. , Fowler M.E. , Kim M. , Bordowitz J. , Katiyar‐Agarwal S. , Vierling E. (2016) Class I and II small heat shock proteins together with HSP101 protect protein translation factors during heat stress. Plant Physiology, 172, 1221–1236. 10.1104/pp.16.00536 27474115 PMC5047077

[plb70064-bib-0056] Meitha K. , Agudelo‐Romero P. , Signorelli S. , Gibbs D.J. , Considine J.A. , Foyer C.H. , Considine M.J. (2018) Developmental control of hypoxia during bud burst in grapevine. Plant, Cell & Environment, 41, 1154–1170. 10.1111/pce.13141 29336037

[plb70064-bib-0057] Middleton W. , Jarvis B.C. , Booth A. (1978) The effects of ethanol on rooting and carbohydrate metabolism in stem cuttings of *Phaseolus aureus* Roxb. New Phytologist, 81, 279–285. 10.1111/j.1469-8137.1978.tb02633.x

[plb70064-bib-0058] Park C.J. , Seo Y.S. (2015) Heat shock proteins: a review of the molecular chaperones for plant immunity. Plant Pathology Journal, 31, 323–333. 10.5423/ppj.rw.08.2015.0150 26676169 PMC4677741

[plb70064-bib-0059] Parsell D.A. , Lindquist S. (1993) The function of heat‐shock proteins in stress tolerance: degradation and reactivation of damaged proteins. Annual Review of Genetics, 27, 437–496. 10.1146/annurev.ge.27.120193.002253 8122909

[plb70064-bib-0060] Patra M. , Salonen E. , Terama E. , Vattulainen I. , Faller R. , Lee B.W. , Holopainen J. , Karttunen M. (2006) Under the influence of alcohol: the effect of ethanol and methanol on lipid bilayers. Biophysical Journal, 90, 1121–1135. 10.1529/biophysj.105.062364 16326895 PMC1367264

[plb70064-bib-0061] Pignataro L. , Miller A.N. , Ma L. , Midha S. , Protiva P. , Herrera D.G. , Harrison N.L. (2007) Alcohol regulates gene expression in neurons via activation of heat shock factor 1. Journal of Neuroscience, 27, 12957–12966. 10.1523/jneurosci.4142-07.2007 18032669 PMC6673276

[plb70064-bib-0062] Plesset J. , Palm C. , McLaughlin C.S. (1982) Induction of heat shock proteins and thermotolerance by ethanol in *Saccharomyces cerevisiae* . Biochemical and Biophysical Research Communications, 108, 1340–1345. 10.1016/0006-291X(82)92147-7 6758774

[plb70064-bib-0063] Raymond P. , Saglio P. , Ricard B. (1995) Réponse au manque d'oxygène dans les tissus végétaux. Cahiers Agricultures, 4, 343–350. https://revues.cirad.fr/index.php/cahiers‐agricultures/article/view/29908/29668

[plb70064-bib-0064] Rolletschek H. , Borisjuk L. , Koschorreck M. , Wobus U. , Weber H. (2002) Legume embryos develop in a hypoxic environment. Journal of Experimental Botany, 53, 1099–1107. 10.1093/jexbot/53.371.1099 11971921

[plb70064-bib-0065] Sako K. , Nguyen H.M. , Seki M. (2020) Advances in chemical priming to enhance abiotic stress tolerance in plants. Plant and Cell Physiology, 61, 1995–2003. 10.1093/pcp/pcaa119 32966567

[plb70064-bib-0066] Sato F. (2013) Characterization of plant functions using cultured plant cells, and biotechnological applications. Bioscience, Biotechnology, and Biochemistry, 77, 1–9. 10.1271/bbb.120759 23291765

[plb70064-bib-0067] Savvides A. , Ali S. , Tester M. , Fotopoulos V. (2016) Chemical priming of plants against multiple abiotic stresses: mission possible? Trends in Plant Science, 21, 329–340. 10.1016/j.tplants.2015.11.003 26704665

[plb70064-bib-0068] Schwacke R. , Ponce‐Soto G.Y. , Krause K. , Bolger A.M. , Arsova B. , Hallab A. , Gruden K. , Stitt M. , Bolger M.E. , Usadel B. (2019) MapMan4: a refined protein classification and annotation framework applicable to multi‐omics data analysis. Molecular Plant, 12, 879–892. 10.1016/j.molp.2019.01.003 30639314

[plb70064-bib-0069] Scott B.R. (2008) Low‐dose‐radiation stimulated natural chemical and biological protection against lung cancer. Dose‐Response, 6, 299–318. 10.2203/dose-response.07-025.scott 18846259 PMC2564762

[plb70064-bib-0070] Smetanska I. (2008) Production of secondary metabolites using plant cell cultures. Advances in Biochemical Engineering/Biotechnology, 111, 187–228. 10.1007/10_2008_103 18594786

[plb70064-bib-0071] Sun Y. , MacRae T.H. (2005) Small heat shock proteins: molecular structure and chaperone function. Cellular and Molecular Life Sciences, 62, 2460–2476. 10.1007/s00018-005-5190-4 16143830 PMC11138385

[plb70064-bib-0072] Todaka D. , Quynh D.T.N. , Tanaka M. , Utsumi Y. , Utsumi C. , Ezoe A. , Takahashi S. , Ishida J. , Kusano M. , Kobayashi M. , Saito K. (2024) Application of ethanol alleviates heat damage to leaf growth and yield in tomato. Frontiers in Plant Science, 15, 1325365. 10.3389/fpls.2024.1325365 38439987 PMC10909983

[plb70064-bib-0073] Triantaphylides C. , Nespoulous L. , Chervin C. (1993) Ammonium requirement for radiation‐induced accumulation of polyamines in suspension‐cultured grape cells. Physiologia Plantarum, 87, 389–395. 10.1111/j.1399-3054.1993.tb01746.x

[plb70064-bib-0074] Tsvetkova N.M. , Horváth I. , Török Z. , Wolkers W.F. , Balogi Z. , Shigapova N. , Crowe L.M. , Tablin F. , Vierling E. , Crowe J.H. , Vigh L. (2002) Small heat‐shock proteins regulate membrane lipid polymorphism. Proceedings of the National Academy of Sciences, 99, 13504–13509. 10.1073/pnas.192468399 PMC129703 12368478

[plb70064-bib-0075] Ul H.S. , Khan A. , Ali M. , Khattak A.M. , Gai W.X. , Zhang H.X. , Wei A.M. , Gong Z.H. (2019) Heat shock proteins: dynamic biomolecules to counter plant biotic and abiotic stresses. International Journal of Molecular Sciences, 20, 5321. 10.3390/ijms20215321 31731530 PMC6862505

[plb70064-bib-0076] Vierling E. (1991) The roles of heat shock proteins in plants. Annual Review of Plant Physiology and Plant Molecular Biology, 42, 579–620. 10.1146/annurev.pp.42.060191.003051

[plb70064-bib-0077] Vitrac X. , Larronde F. , Krisa S. , Decendit A. , Deffieux G. , Mérillon J.M. (2000) Sugar sensing and Ca2+−calmodulin requirement in *Vitis vinifera* cells producing anthocyanins. Phytochemistry, 53, 659–665. 10.1016/s0031-9422(99)00620-2 10746878

[plb70064-bib-0078] Wang B. , Lin L. , Yuan X. , Zhu Y. , Wang Y. , Li D. , He J. , Xiao Y. (2023) Low‐level cadmium exposure induced hormesis in peppermint young plant by constantly activating antioxidant activity based on physiological and transcriptomic analyses. Frontiers in Plant Science, 14, 1088285. 10.3389/fpls.2023.1088285 36755692 PMC9899930

[plb70064-bib-0079] Wang W. , Vinocur B. , Shoseyov O. , Altman A. (2004) Role of plant heat‐shock proteins and molecular chaperones in the abiotic stress response. Trends in Plant Science, 9, 244–252. 10.1016/j.tplants.2004.03.006 15130550

[plb70064-bib-0080] Waters E.R. (2013) The evolution, function, structure, and expression of the plant sHSPs. Journal of Experimental Botany, 64, 391–403. 10.1093/jxb/ers355 23255280

[plb70064-bib-0081] Waters E.R. , Vierling E. (2020) Plant small heat shock proteins–evolutionary and functional diversity. New Phytologist, 227, 24–37. 10.1111/nph.16536 32297991

[plb70064-bib-0082] Wu Z. , Yang L. , Jiang L. , Zhang Z. , Song H. , Rong X. , Han Y. (2019) Low concentration of exogenous ethanol promoted biomass and nutrient accumulation in oilseed rape (*Brassica napus* L.). Plant Signaling & Behavior, 14, 1681114. 10.1080/15592324.2019.1681114 31642378 PMC6866684

[plb70064-bib-0083] Xiao Z. , Rogiers S.Y. , Sadras V.O. , Tyerman S.D. (2018) Hypoxia in grape berries: the role of seed respiration and lenticels on the berry pedicel and the possible link to cell death. Journal of Experimental Botany, 69, 2071–2083. 10.1093/jxb/ery039 29415235 PMC6018838

[plb70064-bib-0084] Zhang J.H. , Huang W.D. , Liu Y.P. , Pan Q.H. (2005) Effects of temperature acclimation pretreatment on the ultrastructure of mesophyll cells in young grape plants (*Vitis vinifera* L. cv. Jingxiu) under cross‐temperature stresses. Journal of Integrative Plant Biology, 47, 959–970. 10.1111/j.1744-7909.2005.00109.x

