# Supplementary material for: Responses of grapevine cells to physiological doses of ethanol, including induced resistance to heat stress
Source: Plant Biol (Stuttg). 2025 Jun 16;27(6):1187–201. doi: 10.1111/plb.70064 (PMC12477316; doi:10.1111/plb.70064)
Supplement: Supplementary file 1 — Fig. S1. Heat stress monitoring in Gamay grape cell cultures. Fig. S2. Average weight of a callus of Vitis vinifera cv. Gamay as a function of development time after sub‐culturing. Fig. S3. Dissolved O2 profiles of Vitis vinifera L. cv. Gamay cell cultures as a function of development time. Fig. S4. Photographs of calli from above and photographs of calli cross‐sections. Fig. S5. Transcriptional dynamics, showing number of DEGs upregulated and downregulated, 6 and 24 h after a 1 mM ethanol treatment, compared to controls. Fig. S6. Details of the 16 DEGs within the enriched GO term “Secondary metabolism process”. Fig. S7. (A and B) Monitoring anthocyanin concentration and total polyphenol index of Gamay cell cultures after an initial 1 mM ethanol treatment or control treatment. Table S1. Composition of the Gamay cell culture growth medium. Table S2. The ‘concentration‐effect’ of ethanol treatment in 3‐week‐old calli, at the time of treatment with exogenous ethanol. Table S3. (A and B) Quality of alignment and Assignment rate to features (RNA‐seq data). [file PLB-27-1187-s006.docx]

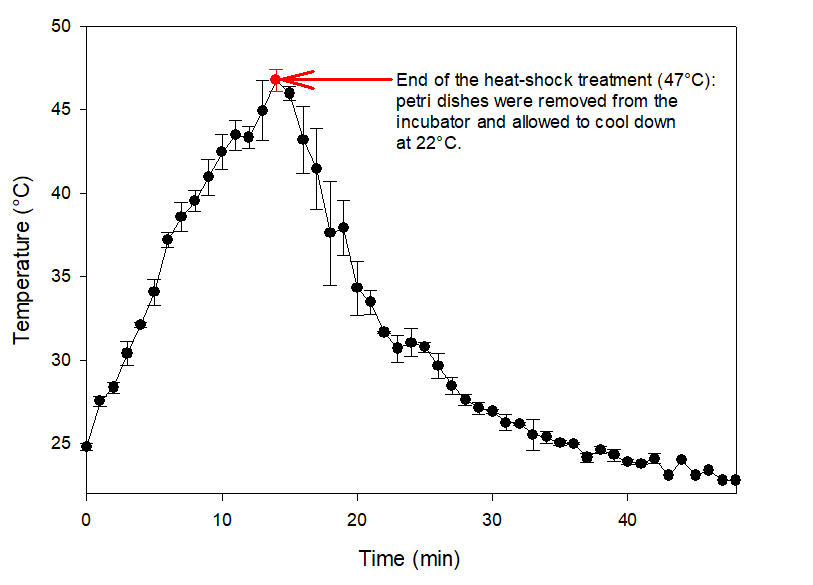

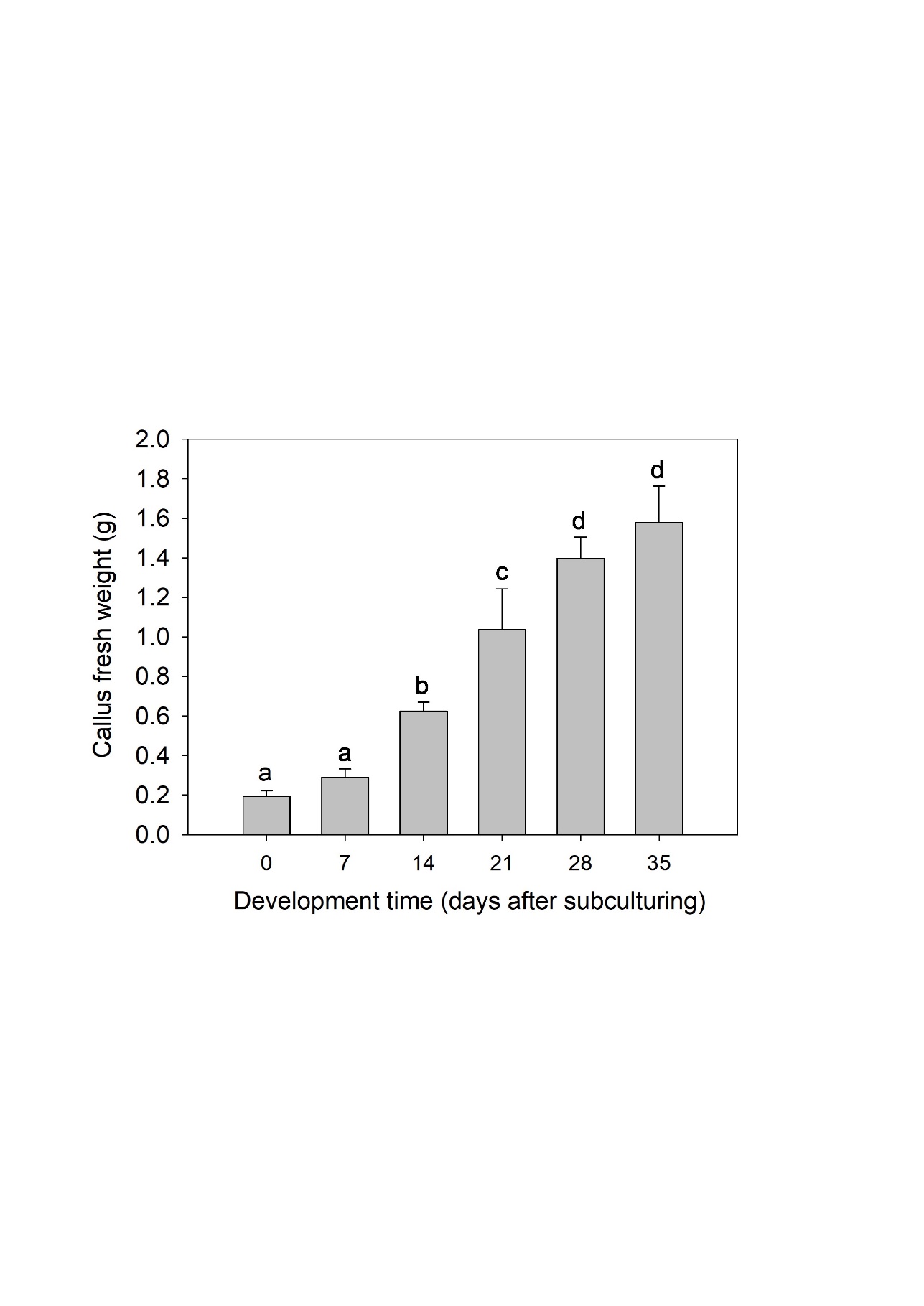


**SuppFig. S1**. Heat-stress monitoring in Gamay cell cultures. The heat stress was applied in an incubator under dark conditions. The data represent the average temperature ± SD at the surface of the growth medium (n=3).

**Supp. Fig. S2**. Average weight of a callus of *Vitis vinifera* cv. Gamay as a function of development time after sub-culturing. n = 12 different calli, error bars show SD, different small letters indicate significant differences at 0.05 (Dunn's test).


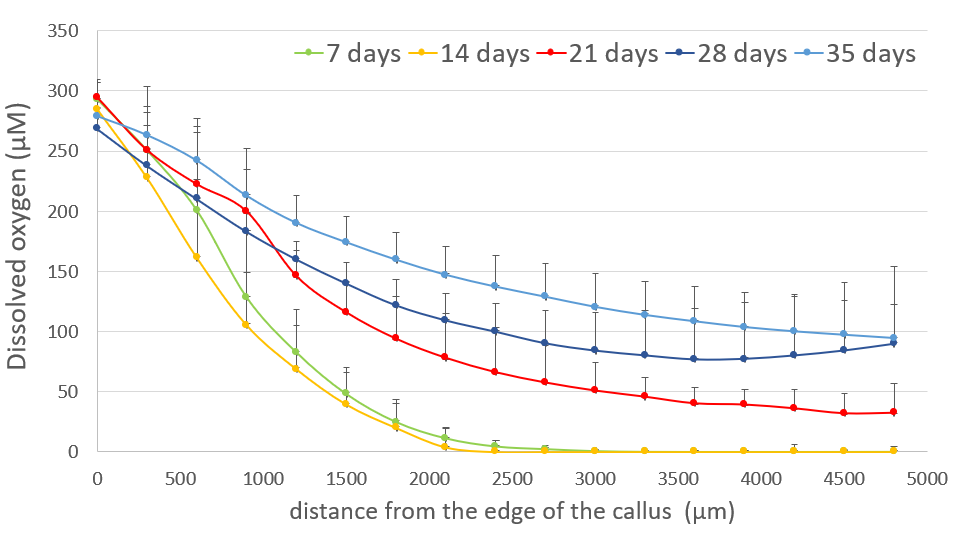


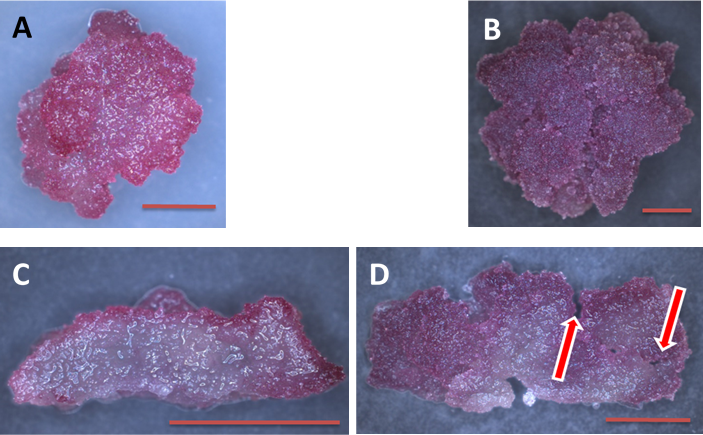


**Supp. Fig. S4**. Photographs of calli seen from above (**A** and **B**) and photographs of calli cross-sections (**C** and **D**). Calli are either 14 days (**A** and **C**) or 35 days (**B** and **D**) old for comparison. Scale: the orange bar represents 0.5 cm. Red arrows show invaginations and gaseous interstices.

**Supp. Fig. S3.** Dissolved O_2_ profiles of *Vitis vinifera* L. cv. Gamay Fréaux cell cultures as a function distance from the edge of the callus (in µm) and the development time (7, 14, 21, 28 and 35 days), n = 4 different calli, error bars show SD.


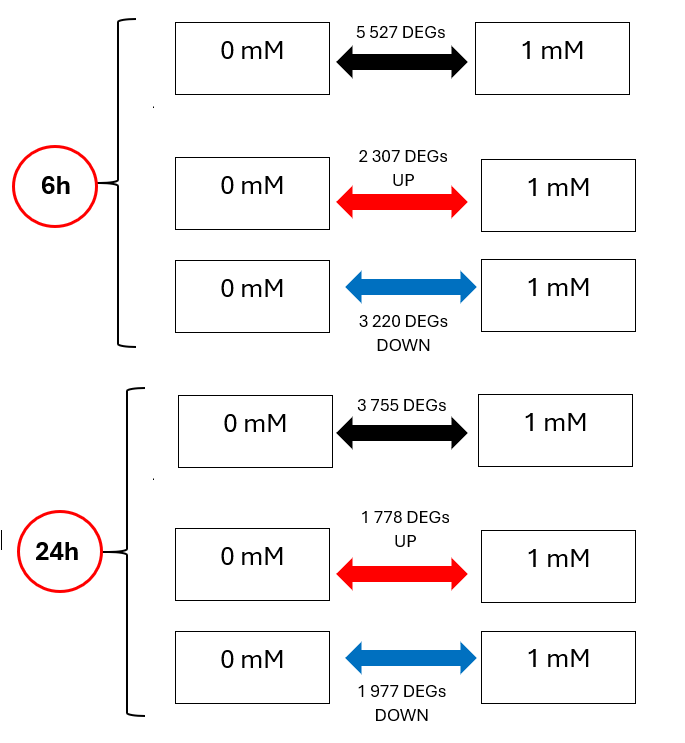

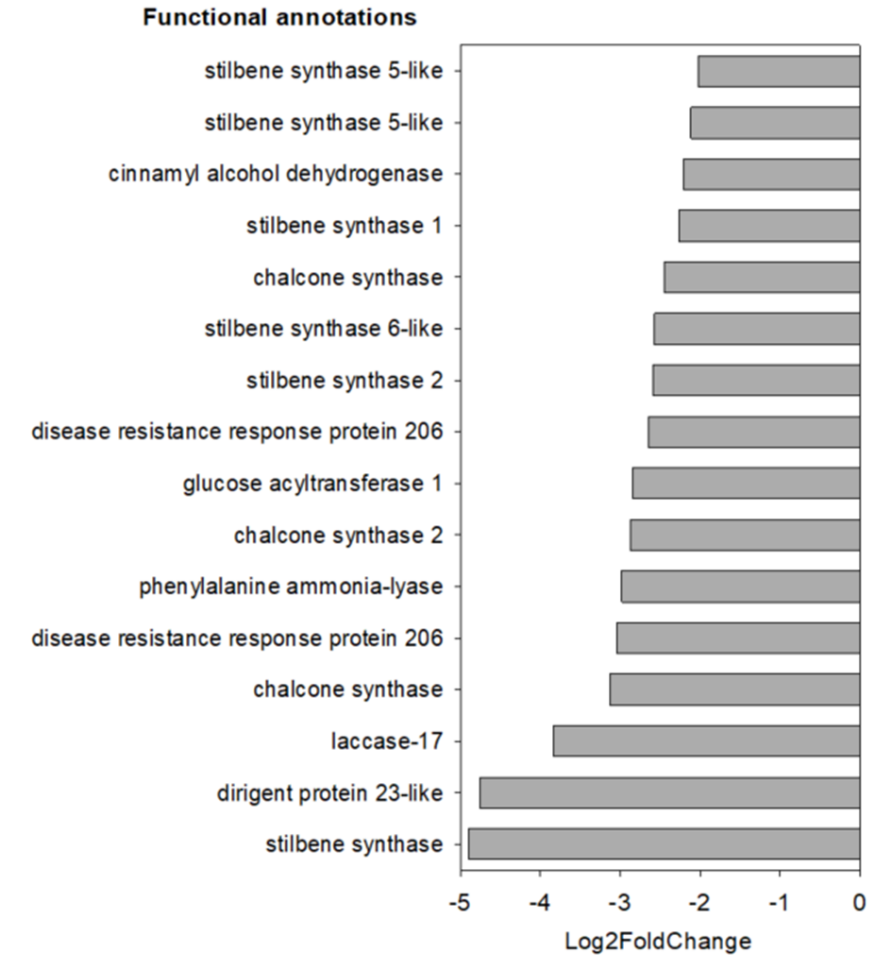


**Supp. Fig. S5**. Transcriptional dynamics showing the number of DEGs upregulated (red arrows) and downregulated (blue arrows), 6 and 24 hours after a 1 mM ethanol treatment, compared to the controls. Black arrows show the total numbers of DEGs. DEGs shown in this figure are all the genes with a p-adj < 0.05, with no regard to their Log2FoldChange.

**Supp. Fig. S6**. Details of the 16 DEGs, within the enriched GO term "secondary metabolism process", ordered by their respective fold changes, comparing 1 mM ethanol treatment to control, at 6 hour post-treatment.


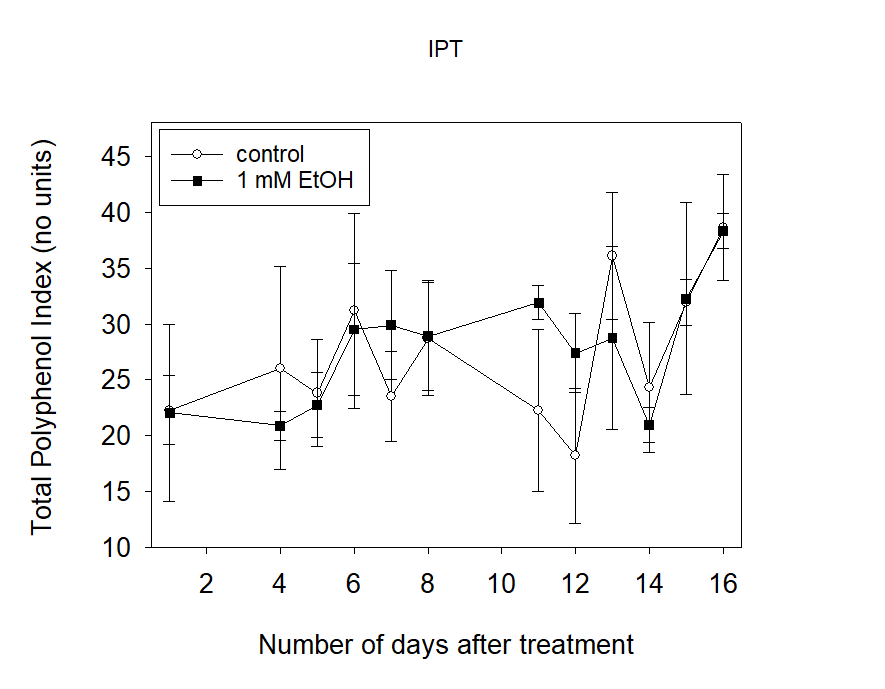


**B**

**A**


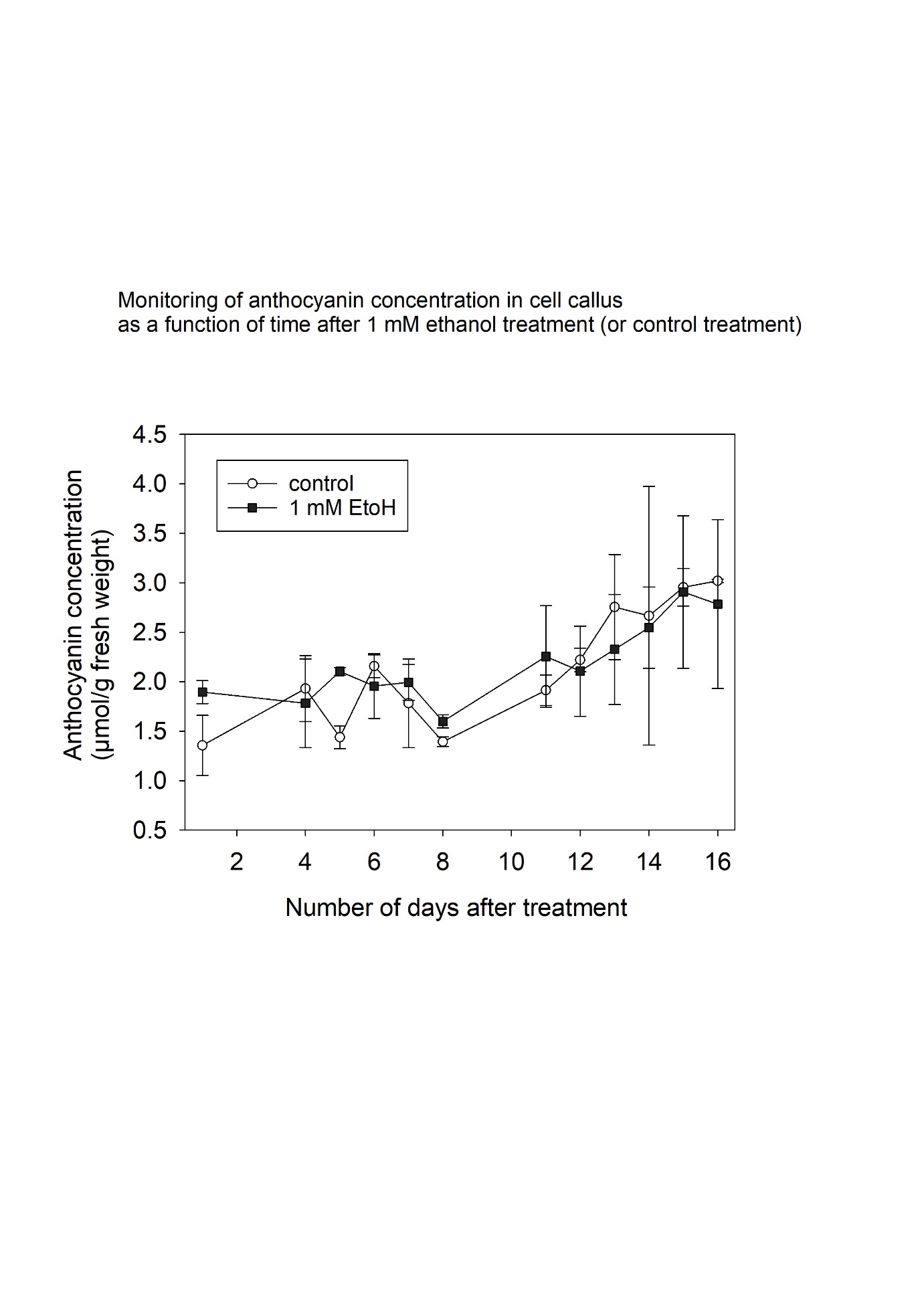


**Supp. Fig. S7**: Temporal changes in **A)** anthocyanin concentration and **B)** in the total polyphenol index (TPI) in grapevine cell cultures following an initial treatment of 1 mM ethanol (black squares) or a control treatment (white circles). Data represent the mean ± SD of three samples.

**Supp. Table S1.** Composition of growth medium for *Vitis vinifera* Gamay Fréaux cell cultures *(as it is not easy to find in old articles)*


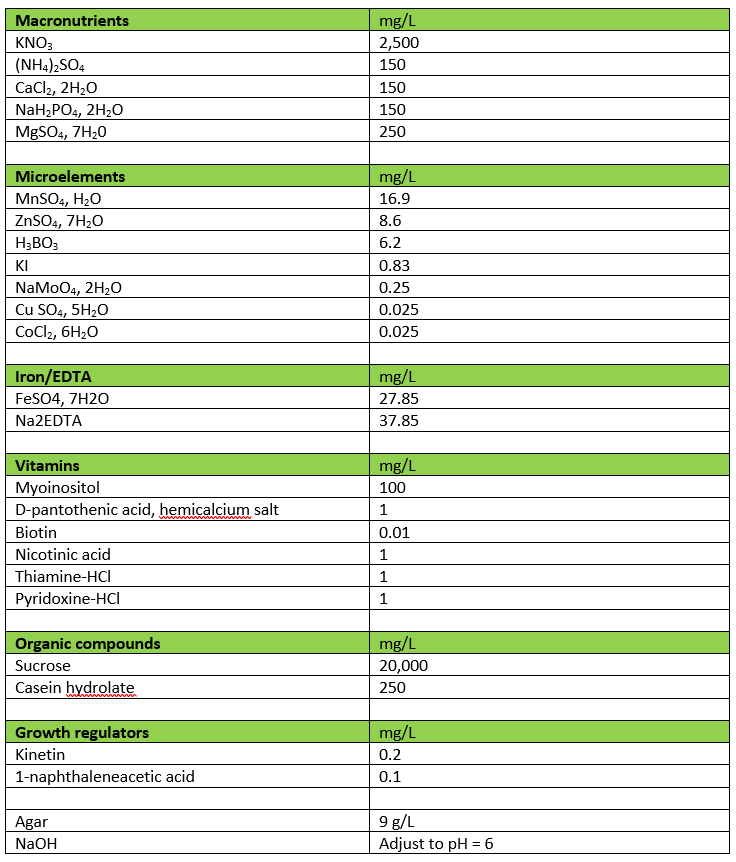


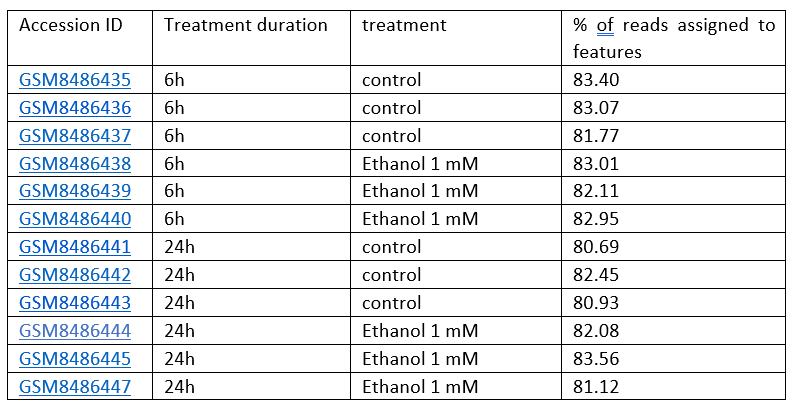

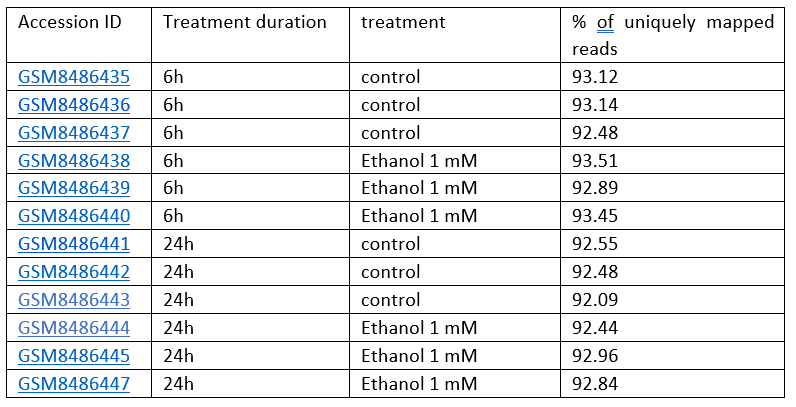

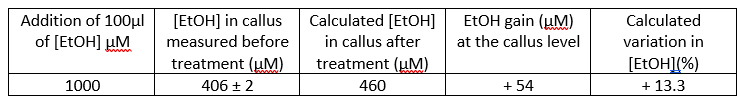


*Supp. Tables S4 to S9 (Excel files) are available as Supp. Data via the link provided line 725 (main ms), as including them led the ScholarOne platform to generate a 1,350-page PDF file.*

**Supp. Table S3B**. Assignment rate to features on the *Vitis vinifera* cv. Chasselas reference genome (Djari *et al*., 2024).

**Supp. Table S3A**. Quality of the alignment - Percentage of uniquely mapped reads on the *Vitis vinifera* cv. Chasselas reference genome (Djari *et al*., 2024). BioProject Number : PRJNA1153273 (accessible through <https://www.ncbi.nlm.nih.gov/bioproject/PRJNA1153273>).

*N.B: The addition of 100 μl of an ethanol solution at 1mM results in a calculated final concentration in the callus of 460 μM, i.e. an increase of +13.3% compared to an untreated callus.*

**Supp. Table S2**. Table showing the ‘concentration-effect’ of the ethanol treatment in 3-week-old calli, at the time of treatment with the exogenous ethanol solution. The endogenous ethanol concentration was measured in 3 calli. The final ethanol concentration after treatment was calculated to account for the light ethanol change generated by the 1 mM EtOH treatment.
